# A Dynamic Gate Enables Regioselective Hydroxylation of Free Arginine by a Non‐Canonical Heme Enzyme

**DOI:** 10.1002/advs.202513032

**Published:** 2025-11-12

**Authors:** Yuan Sun, Chao Dou, Weizhu Yan, Pengpeng Chen, Lu Zhang, Dan Zhou, Yanhui Zheng, Zhaolin Long, Shoujie Li, Xiaoqing Xu, Qiuxia Huang, Xiaofeng Zhu, Wei Cheng

**Affiliations:** ^1^ Department of Pulmonary and Critical Care Medicine Respiratory Infection and Intervention Laboratory of Frontiers Science Center for Disease‐related Molecular Network and State Key Laboratory of Biotherapy West China Hospital of Sichuan University Chengdu 610041 China; ^2^ Antibiotics Research and Re‐evaluation Key Laboratory of Sichuan Province Sichuan Industrial Institute of Antibiotics School of Pharmacy Chengdu University Chengdu 610106 China; ^3^ Metabolomics and Proteomics Technology Platform of Core Facilities West China Hospital Sichuan University Chengdu 610041 China

**Keywords:** crystal structure, dynamic regiochemical gating mechanism, heme‐dependent hydroxylase, self‐sufficient artificial chimeric enzyme, YqcI/YcgG family enzymes

## Abstract

The YqcI/YcgG family of heme‐dependent enzymes catalyzes guanidine N–H hydroxylation, a critical yet enigmatic step in bioactive natural product biosynthesis. Here, this mechanistic puzzle is resolved through high‐resolution structural snapshots of AglA, a prototypical YqcI/YcgG member, revealing a non‐canonical heme‐binding “sandwich” fold. A dynamic regiochemical gating mechanism is uncovered: substrate‐induced remodeling of loop L2 and key residues (Phe152, Arg179, Phe182) spatially constrains the guanidine group of aminomethylphosphonate‐linked arginine (AMPn‐Arg), enforcing exclusive internal N^ε^ hydroxylation. Single‐site mutations rewire hydrogen‐bond networks to enable hydroxylation of free L‐arginine with controllable regioselectivity (internal N^δ^ vs terminal N^ω^) while preserving native internal N^ε^ selectivity for AMPn‐Arg. Crystal structures of engineered variants with free arginine, together with MD simulations, explain how subtle rearrangements of loop L2 and residues Phe152/Arg179/Phe182 pivot the guanidinium group relative to the heme Fe(IV) = O intermediate. Fusing AglA to its native PDR/VanB reductase yields a self‐sufficient chimera with improved catalytic efficiency. This work establishes a structural blueprint for tuning guanidino N–H hydroxylation and demonstrates proof‐of‐principle control of regioselectivity in a non‐canonical heme enzyme, thereby advancing the synthesis of arginine‐based antibiotics and precision‐functionalized therapeutics.

## Graphical abstract

The YqcI/YcgG enzyme AglA employs a unique “sandwich” fold and a dynamic gating mechanism to selectively hydroxylate AMPn‐Arg at N^ε^. Site‐directed mutations reprogram regioselectivity or enable activity on free arginine by altering hydrogen‐bond networks, precisely positioning substrates. This work establishes a programmable biocatalytic platform for synthesizing arginine‐based antibiotics and functionalized therapeutics.

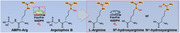

## 1 Introduction

Hydroxylation is a pivotal transformation in natural product biosynthesis that enhances structural diversity and bioactivity by introducing oxygen functional groups.^[^ ^1^, ^2^, ^3^, ^4^, ^5^, ^6^, ^7^, ^8^, ^9^ ^]^ While cytochrome P450 monooxygenases dominate enzymatic C─H bond activation,^[^ ^10^, ^11^, ^12^, ^13^ ^]^ selective N─H hydroxylation, particularly of guanidine groups, remains a formidable challenge owing to the lack of high‐resolution crystal structure and mechanistic understanding.

**Figure 1:**
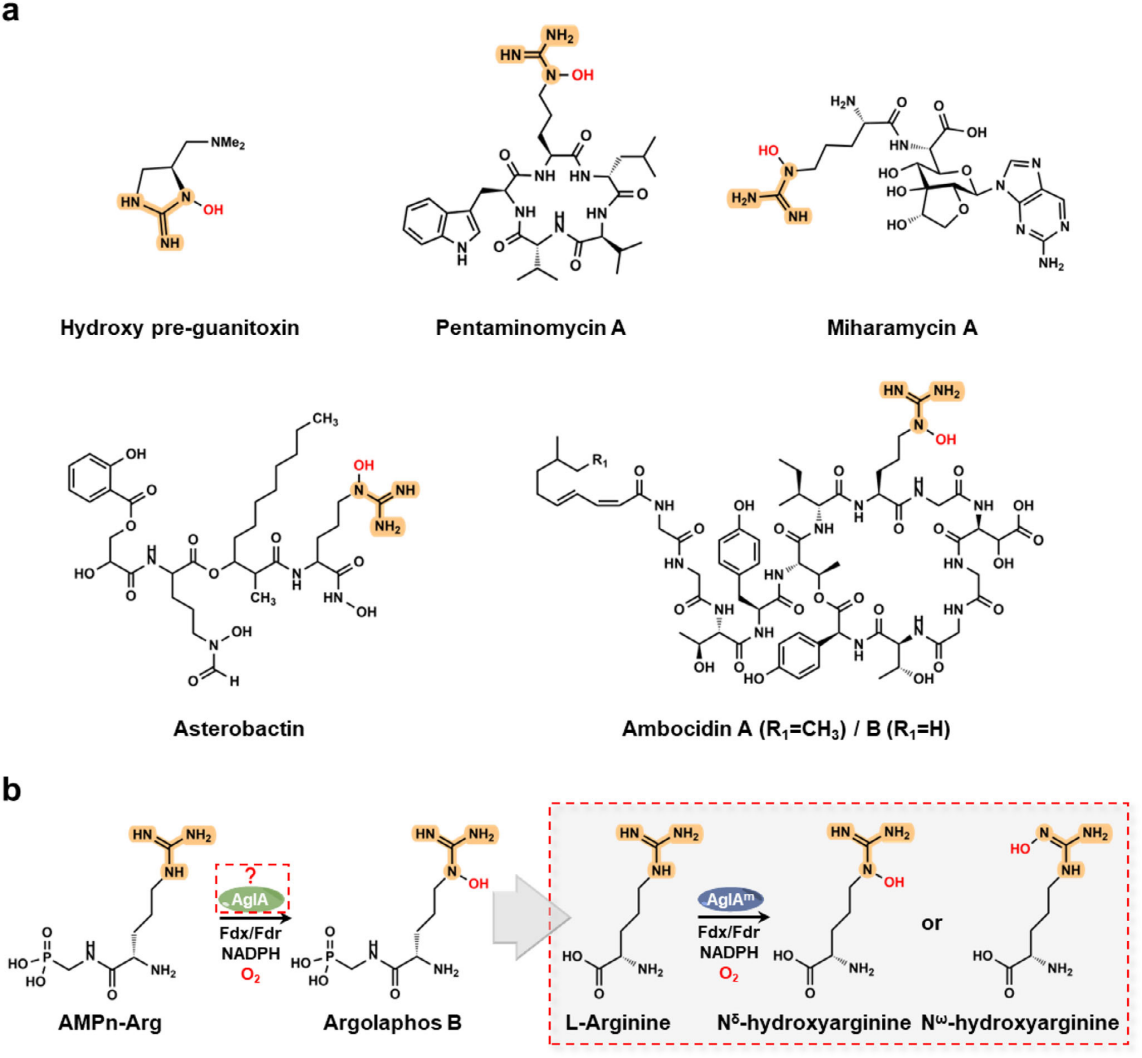
Guanidine hydroxylation in natural product biosynthesis. a) Representative N^ε^‐ or N^ω^‐hydroxyarginine containing natural products. The guanidine group is highlighted, and the hydroxyl is colored red. b) AglA‐catalyzed conversion of AMPn‐Arg to argolaphos B via N^ε^‐hydroxylation. The molecular basis governing regioselectivity and substrate specificity remains unresolved. Engineered AglA variants developed in this study expand functionality by catalyzing the hydroxylation of L‐arginine at either the N^ω^ position (yielding N^ω^‐hydroxyarginine) or the N^δ^ position (yielding N^δ^‐hydroxyarginine).

The YqcI/YcgG family represents a unique class of heme‐dependent enzymes specializing in guanidine N─H hydroxylation, embedded within biosynthetic pathways of antimicrobial agents such as miharamycins,^[^ ^14^ ^]^ ambocidins,^[^ ^15^ ^]^ guanitoxin^[^ ^16^ ^]^ and argolaphos^[^ ^17^ ^]^ (**Figure** **1**a). Despite their critical role in producing bioactive metabolites, the structural and mechanistic basis for their regioselectivity and substrate specificity has remained elusive, hindering their exploitation as biocatalysts. YqcI/YcgG enzymes are ubiquitous across all domains of life, yet only six bacterial members have been functionally characterized^[^ ^14^, ^15^, ^16^, ^17^, ^18^, ^19^, ^20^, ^21^, ^22^ ^]^ (Figure S1, Supporting Information). These enzymes catalyze hydroxylation at distinct positions (internal or terminal N) of arginine derivatives during natural product biosynthesis^[^ ^14^, ^15^, ^16^, ^17^, ^19^, ^22^ ^]^ (Figure S2, Supporting Information), but atomic‐level details of their catalytic mechanisms are absent. AglA, the archetype of this family, mediates internal N^ε^‐hydroxylation of aminomethylphosphonate‐linked arginine (AMPn‐Arg), a key step in the synthesis of argolaphos B, a phosphonopeptide with potent activity against *Salmonella typhimurium*, *Escherichia coli*, and *Staphylococcus aureus*.^[^ ^17^, ^20^ ^]^ Nevertheless, the stringent regioselectivity and failure of the enzyme to act on free arginine reveal lingering ambiguities in its substrate recognition principles and catalytic machinery.

Here, we integrate crystallography, biochemistry, computational modeling, and enzyme engineering to decipher the catalytic logic of AglA. We reveal a dynamic regiochemical gating mechanism enforced by conformational remodeling of loop L2 and key residues, which spatially constrains the guanidine group for N^ε^‐specific hydroxylation. Through rational mutagenesis, we enable AglA to hydroxylate free arginine at either internal N^δ^ or terminal N^ω^, resulting in unprecedented catalytic plasticity. Furthermore, we engineered a self‐sufficient AglA‐reductase chimera by tethering the redox partner to AglA, thereby facilitating efficient electron transfer and significantly enhancing turnover. These findings not only resolve long‐standing questions about enzymatic N–H activation but also establish YqcI/YcgG enzymes as tunable platforms for controlling guanidino N–H hydroxylation in synthesizing arginine‐based therapeutics.

## 2 Results

### 2.1 Unique Heme‐Bound Architecture of AglA

**Figure 2:**
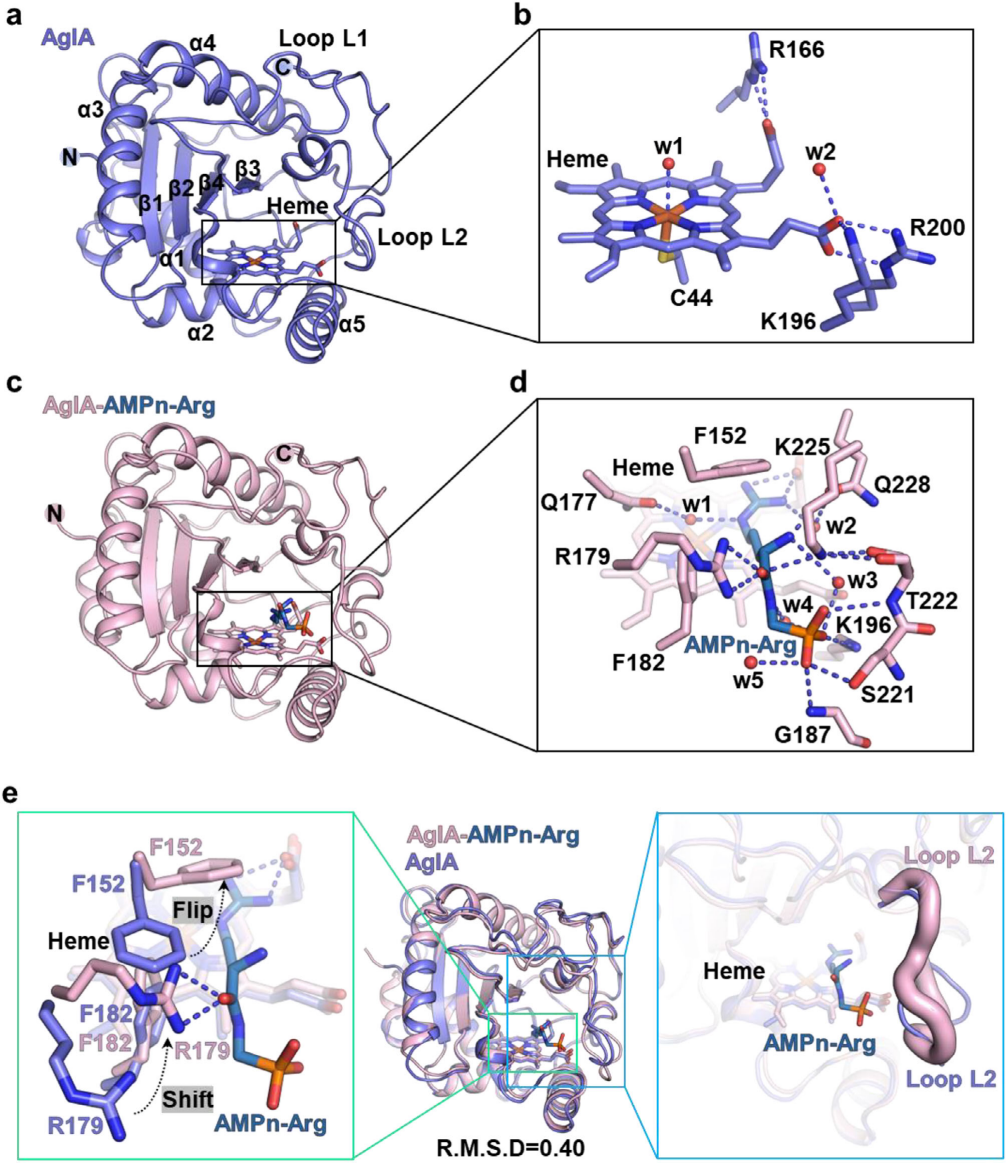
Structural basis for the catalytic mechanism of AglA. a) Overall structure of apo AglA, showing its unique “sandwich” fold with a central β‐sheet flanked by helices and loops. b) Heme coordination in apo AglA. The heme iron is ligated by Cys44 thiolate, with carboxylate groups forming salt bridges (dashed blue lines) with Arg166, Lys196, and Arg200; a water molecule (red sphere) mediates hydrogen bonding. c) Structure of the AglA‐AMPn‐Arg complex. AMPn‐Arg is located at the distal axial side of the heme within the catalytic cavity. d) Detailed interactions between AglA active site residues and AMPn‐Arg. The substrate forms a comprehensive network of ionic interactions, hydrogen bonds (dashed blue lines), and hydrophobic contacts with surrounding residues and water molecules. e) Substrate‐induced conformational changes. Superposition of AMPn‐Arg‐bound and apo AglA structures (RMSD = 0.40 Å, middle) reveals significant side chain rearrangements of key residues (Phe152, Arg179, and Phe182, green box) and conformational shifts in loop L2 (blue box). Loop L2 is depicted in tube form, with the thickness reflecting the atomic displacement parameters (B factors).

To investigate the catalytic mechanism of AglA, we determined its crystal structure in the heme‐bound (apo) state at 2.1 Å resolution (Table S1, Supporting Information). AglA adopts a sandwich‐like architecture, featuring a central four‐stranded β‐sheet (β1–β4) flanked by three helices (α1, α2, α5) on one side and two helices (α3, α4) on the opposite side. Two extended loops, L1 (connecting α4 and β3) and L2 (extending from α5 to the C‐terminus), form a boundary around one edge of the β‐sheet near β3 (**Figure** **2**a; Figure S3a, Supporting Information). The heme cofactor is positioned between the three‐helix face and the β‐sheet, with loop L2 forming a catalytic cavity bordered by β3, β4, and itself (Figure 2a; Figure S3b, Supporting Information). The heme iron is axially coordinated by the thiol group of Cys44, and its carboxylate groups form stabilizing salt bridges with Arg166, Lys196, and Arg200 (Figure 2b).

A DALI search^[^ ^23^ ^]^ revealed no significant similarity between AglA and previously characterized heme enzymes, in which the DALI top hits (Z  score≤ 5.4; Table S2, Supporting Information) share only partial topology, highlighting AglA as a new structural class. Although AglA lacks the α‐helical core and PROSITE motif typical of class I P450 monooxygenases,^[^ ^24^, ^25^ ^]^ its thiolate‐ligated heme cofactor mirrors the general P450 hallmark of cysteine coordination. AlphaFold3^[^ ^26^ ^]^ modeling of five additional YqcI/YcgG family members (DcsA, ChmN, AmbT, GntA, and Mhr24) confirmed a conserved sandwich fold and cysteine coordination (Figure S4a,b, Supporting Information), identifying AglA as a prototypical representative. These insights reveal an unrecognized repertoire of thiolate‐ligated heme enzymes beyond canonical P450s.

### 2.2 Active Site Dynamics and Catalytic Regulation

To understand how AglA distinguishes between its native substrate AMPn‐Arg and inactive unmodified arginine, we solved the high‐resolution crystal structures of AglA complexed with AMPn‐Arg (1.6 Å) and free arginine (1.8 Å) (Table S1, Supporting Information). These structures reveal critical active‐site interactions and substantial conformational changes upon ligand binding, which are essential for substrate specificity and catalysis.

In the AMPn‐Arg complex, the substrate binds to the distal axial side of the heme through a network of ionic, hydrogen bonding and hydrophobic interactions (Figure 2c,d; Figure S5, Supporting Information). Superimposing the AMPn‐Arg–bound structure onto apo‐AglA revealed substantial movement of loop L2 and rearrangements in Phe152, Arg179, and Phe182, cooperatively forming a substrate‐binding pocket for both the AMPn moiety and the arginine backbone (Figure 2e). The AMPn phosphate group engages Lys196, Lys225, and residues from loop L2 (Ser221, Thr222, Gly187) through ionic and hydrogen bonding. Additionally, Arg179 stabilizes the backbone carbonyl of the substrate, and Gln228 forms hydrogen bonds with the backbone amino group via a bridging water molecule (w2) (Figure 2d). Phe152 and Phe182 rearrange to sandwich the arginine alkyl side chain, whereas the guanidine group electrostatically interacts with the heme carboxylate, orienting N^ε^ toward the heme distal site (Figure 2d). A bridging water molecule, hydrogen bonded to Gln177, positions the guanidine for selective oxidation at the N^ε^ atom (Figure 2d). In the AglA–AMPn‐Arg structure, the distance from the heme iron to the N^ε^ atom of AMPn‐Arg is 4.3 Å, and that to the N^ω^ atom is 4.4 Å, suggesting that the N^ε^ atom is closer to the heme iron for the preference of its hydroxylation. The active site is notably spacious and solvent exposed, with ordered water molecules contributing additional stabilizing interactions (Figure 2d).

**Figure 3:**
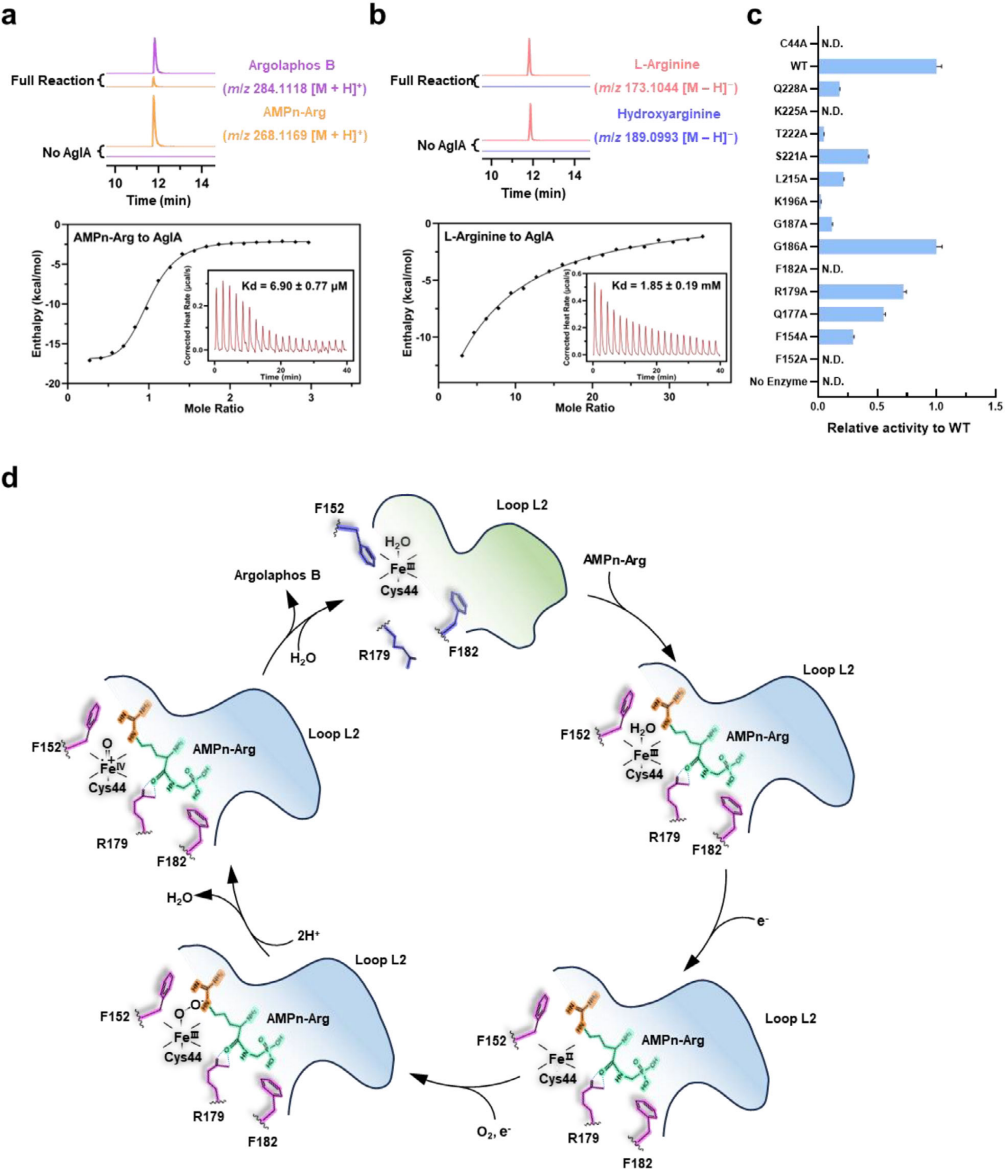
Substrate specificity and gating mechanism in AglA. a) Substrate specificity analysis of AglA toward AMPn‐Arg. LC‒HRMS extracted ion chromatograms showing the conversion of AMPn‐Arg (orange, *m*/*z* 268.1169 [M + H]⁺) to argolaphos B (purple, *m*/*z* 284.1118 [M + H]⁺). Inset: Isothermal titration calorimetry (ITC) reveals the high binding affinity of AMPn‐Arg for AglA. b) AglA activity toward L‐arginine. LC‒HRMS extracted ion chromatograms display L‐arginine (pink, *m*/*z* 173.1044 [M – H]^−^) and negligible hydroxyarginine (slate blue, *m*/*z* 189.0993 [M – H]^−^), with the corresponding ITC inset showing weak binding affinity. c) Relative enzymatic activity of AglA variants. Mutations in key active site residues abolish or reduce catalysis (N.D., not detected; WT, wild type). Data from technical triplicate (n = 3) were used to calculate and display the average ± SD of each variant. SD, standard deviation. Error bars, SD. d) Proposed gating model of AglA. The guanidino groups of AMPn‐Arg (yellow) and the main chain (green) are oriented by loop L2, with conformational changes upon substrate binding shown in light green (before) and light blue (after), and critical side chain rearrangements of Phe152, Arg179, and Phe182 are depicted in slate blue (before binding) and purple (after binding), enabling N^ε^‐hydroxylation.

In contrast, the structure of AglA with free arginine lacks these stabilizing interactions. Loop L2 remains in a conformation resembling the apo state, and residues such as Lys225, Lys196, and Arg179 fail to form polar contacts with the arginine backbone (Figure S6, Supporting Information). Consequently, the arginine backbone adopts an opposite orientation to that of AMPn‐Arg, and the guanidine group rearranges so that N^ω^ faces the heme with the distance of 3.1 Å to heme iron and occupies the space directly above the heme iron, which sterically hinders the binding of oxygen to the heme iron, thereby preventing the productive hydroxylation (Figure S6d, Supporting Information). Consistently, wild‐type AglA exhibited high catalytic efficiency for AMPn‐Arg but was essentially inactive for free arginine (**Figure** **3**a,b).

Site‐directed mutagenesis corroborated the importance of these interactions (Figure 3c; Figure S7, Supporting Information). Mutations in the heme‐coordinating Cys44 completely abolished the catalytic activity, highlighting the essential role of heme coordination. Similarly, substitutions of Phe152 and Phe182 with alanine abolished hydroxylation, validating their role in substrate alignment. Mutations in loop L2 significantly impaired turnover, emphasizing the importance of loop rearrangements in orienting both the substrate backbone and the guanidine group. Together, these findings reveal a dynamic regiochemical gating mechanism in which loop L2, Arg179, and Phe152/Phe182 facilitate substrate backbone fixation and the precise orientation of the guanidine N^ε^ atom to the heme center, enabling N^ε^‐specific hydroxylation (Figure 3d). Although the Fe(IV) = O intermediate was not captured, we speculate that AglA catalyzes AMPn‐Arg via a similar electron transfer pathway based on analogous enzymes.^[^ ^27^, ^28^, ^29^ ^]^

### 2.3 Engineering Substrate Scope Through Active‐Site Mutagenesis

**Figure 4:**
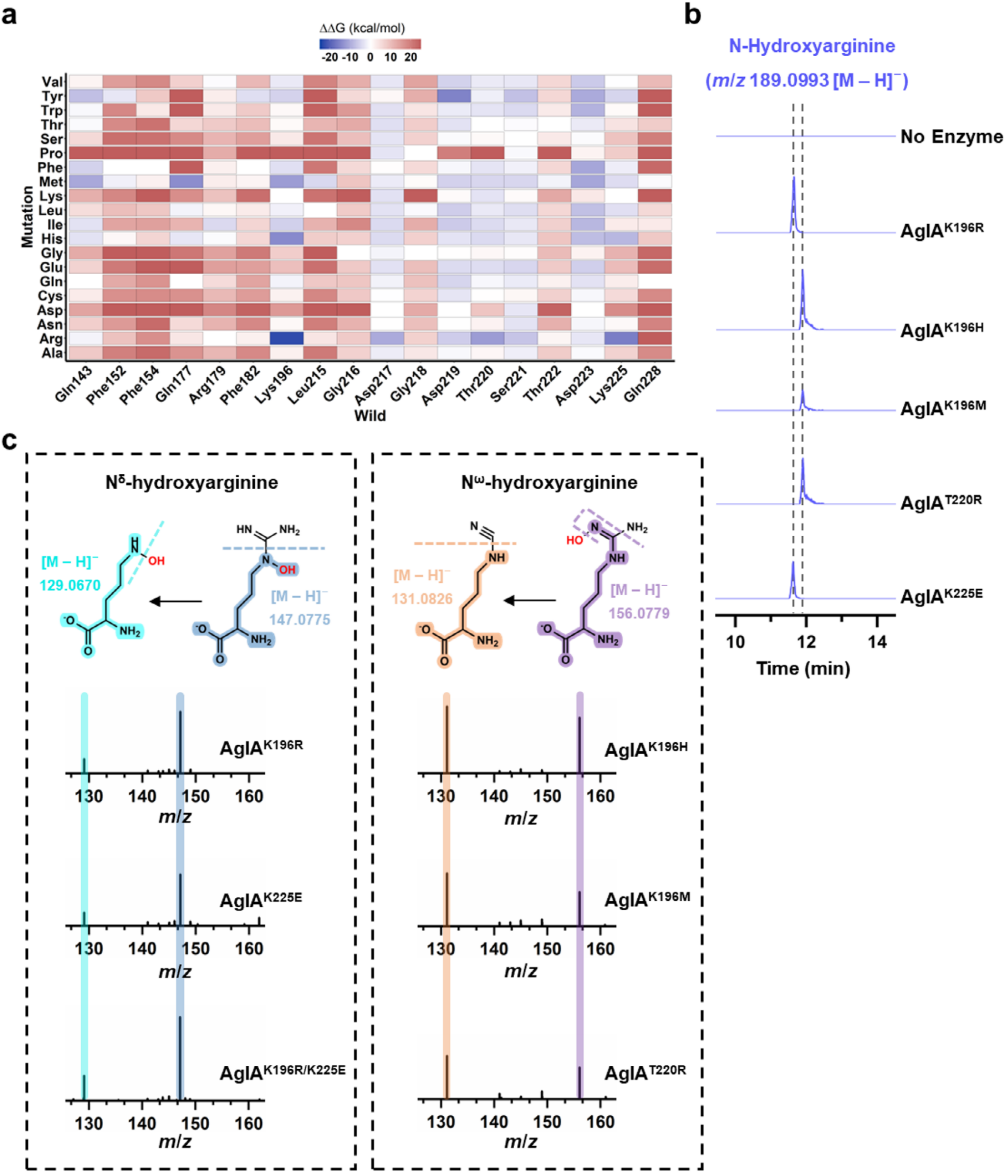
Rational engineering of the regioselectivity of AglA. a) Computational design approach for AglA engineering. Virtual saturation mutagenesis was performed with energy calculations to identify promising variants for arginine hydroxylation. b) LC‒HRMS analysis of engineered AglA variant activity toward free arginine. The extracted ion chromatograms show N‐hydroxyarginine products (*m*/*z* 189.0993 [M – H]^−^), with distinct retention times (black dotted lines), indicating regioselective differences. c) LC‒HRMS/MS confirmation of hydroxylation regioselectivity. Diagnostic fragment ions at *m*/*z* 129.0670 and 147.0775 [M – H]^−^ verify N^δ^‐hydroxylation (left panel), whereas fragments at *m*/*z* 131.0826 and 156.0779 [M – H]^−^ confirm N^ω^‐hydroxylation (right panel) for variant‐specific products.

Despite binding to free arginine, AglA fails to induce a catalytically reactive conformation (Figure 3b; Figure S6, Supporting Information). To explore whether active‐site mutations could reposition free arginine for catalysis, we conducted computational saturation mutagenesis of eighteen residues within the substrate‐binding pocket, including loop L2 and the adjacent arginine‐binding region (**Figure** **4**a). Eight single mutants (K196R, K196M, K196H, D219Y, T220R, D223F, Q177M, and K225R) were selected for experimental validation, together with a charge‐reversal mutant (K225E) to investigate whether altering local electrostatics could promote a catalytically favorable arginine orientation.

Notably, mutations at Lys196 (K196R, K196M, K196H), Thr220 (T220R), and Lys225 (K225E) enabled AglA to hydroxylate free L‐arginine, as evidenced by a *m*/*z* 189.0993 peak in the LC‒HRMS spectra corresponding to hydroxylated arginine (Figure 4b; Figure S8a, Supporting Information). The K196R/K225E double mutant further enhanced this activity, and its significantly higher *k*_cat_/*K*_m_ value compared with that of the single mutant suggests a synergistic effect of the combined mutations on enhancing catalytic efficiency (Figure S8b, Table S3, Supporting Information). Notably, two distinct retention times of the hydroxylated products were observed via LC‒HRMS, indicating two distinct hydroxylation patterns, corresponding to different conformations of arginine in the mutated active sites (Figure 4b). Tandem LC–HRMS/MS analysis^[^ ^16^ ^]^ confirmed that K196R, K225E, and K196R/K225E hydroxylate at the N^δ^ position, whereas K196H, K196M, and T220R preferentially target the N^ω^ position (Figure 4c). Importantly, all the mutants retained native regioselectivity toward AMPn‐Arg (Figure S9, Supporting Information), demonstrating that the extensive interactions of the AMPn moiety remain dominant for that substrate.

### 2.4 Structural Basis for Regioselectivity Switching

**Figure 5:**
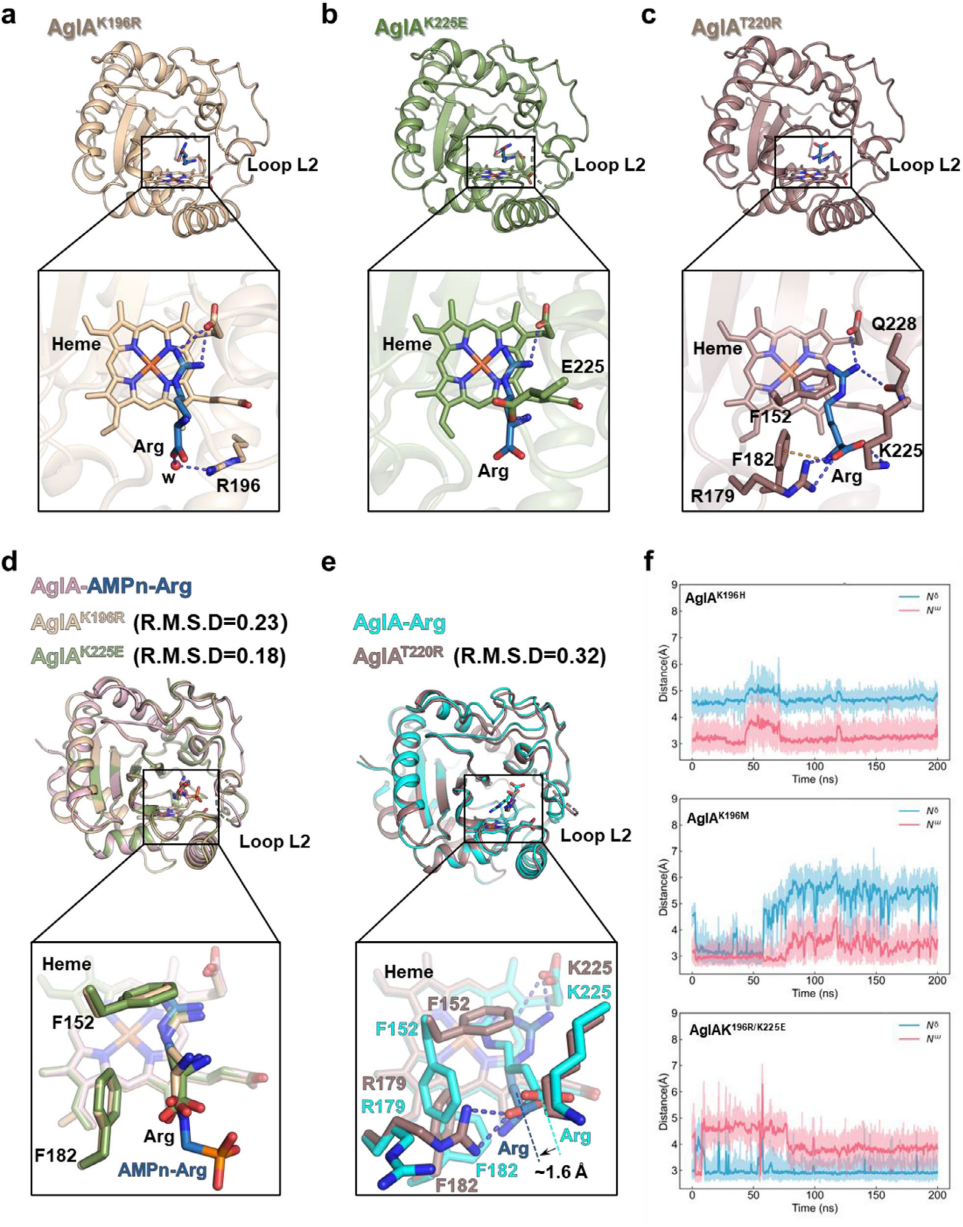
Structural and dynamic insights into regiochemical switching. a) AglA^K196R^‐Arg complex. Key interactions (black box) between arginine and active‐site residues, with a water molecule (“w”, red sphere), stabilize the N^δ^‐facing orientation. b) AglA^K225E^‐Arg complex. Active site interactions (black box) anchor arginine for N^δ^‐hydroxylation. c) AglA^T220R^‐Arg complex. An alternative binding mode (black box) repositions arginine for N^ω^‐hydroxylation. d) Structural overlay of AglA‐AMPn‐Arg, AglA^K196R^‐Arg and AglA^K225E^‐Arg, with RMSD values relative to those of the AglA‐AMPn‐Arg structure. The key residues stabilizing the orientation of the substrates are highlighted. e) Structural comparison of AglA‐Arg and AglA^T220R^‐Arg. Ionic interactions and an ≈1.6 Å backbone shift in T220R (indicated) alter guanidine positioning for N^ω^‐hydroxylation. f) Molecular dynamics (MD) simulations of engineered AglA variants. The graphs track the evolution of distances between the arginine N^ω^ (red lines) and N^δ^ (blue lines) atoms relative to the oxygen atom of the Fe(IV)=O catalytic intermediate over the simulation time. The results are shown for AglA^K196H^ (top), AglA^K196M^ (middle), and AglA^K196R/K225E^ (bottom), illustrating how mutations alter the position of different nitrogen atoms relative to the reactive oxygen species, thereby determining regioselectivity.

To elucidate the structural basis for these altered hydroxylation patterns, we solved the crystal structures of three representative mutants—K196R, K225E, and T220R—each bound to free arginine (Figure S10, Table S4, Supporting Information). In the K196R and K225E mutants, which both favor N^δ^‐hydroxylation, the arginine side chain closely resembles the AMPn‐Arg conformation observed in wild‐type AglA (**Figure** **5**d). The guanidine N^δ^ faces the heme's distal axis, whereas N^ω^ engages in ionic interactions with the heme carboxylate, reducing its reactivity (Figure 5a,b). Although loop L2 is partially disordered in these variants, the longer basic side chain in K196R forms a water‐bridged interaction with the arginine carboxylate, and the newly introduced glutamate in K225E anchors the amino group (Figure 5a,b). These interactions stabilize the arginine backbone in a conformation similar to that of AMPn‐Arg, with overlapping carbonyl atoms (Figure 5d). Despite minor shifts in Cα, the guanidine orientation remains conducive to N^δ^‐hydroxylation. Similarly, Phe152 and Phe182 adopt rotamers akin to those in the AMPn‐Arg complex, maintaining an N^δ^‐facing conformation (Figure 5d).

In contrast, the T220R mutation disrupts loop L2, leaving the mutation site invisible in the electron density map. However, the arginine backbone remains oriented similarly to that of wild‐type AglA but in an orientation opposite that of K196R and K225E (Figure 5c,e). T220R induces shifts in Lys225 and Arg179, which maintain ionic interactions with the arginine carboxylate, causing the backbone to shift ≈1.6 Å closer to Phe182 (Figure 5e). This shift forces local rearrangements in Phe152 and Phe182, altering the side‐chain conformation. Consequently, the guanidine group is repositioned closer to Gln228 and the heme carboxylate, positioning N^ω^ near the reactive iron–oxo species for N^ω^‐hydroxylation while moving N^δ^ too far for productive catalysis (Figure 5e).

Further confirmation of these structural insights was obtained through 200 ns molecular dynamics (MD) simulations with an Fe(IV) = O intermediate, analogous to the reactive species in P450s.^[^ ^30^, ^31^ ^]^ Variants favoring N^ω^‐hydroxylation (K196H, K196M) consistently presented shorter N^ω^–O distances, whereas K196R/K225E mutants presented persistently shorter N^δ^–O distances (Figure 5f; Figure S11, Supporting Information). Together, these results underscore how small residue changes can pivot the guanidine orientation of free arginine, conferring distinct regioselectivities while leaving recognition of AMPn‐Arg largely unaffected.

### 2.5 Development of a Self‐Sufficient AglA‐Reductase Chimera

**Figure 6:**
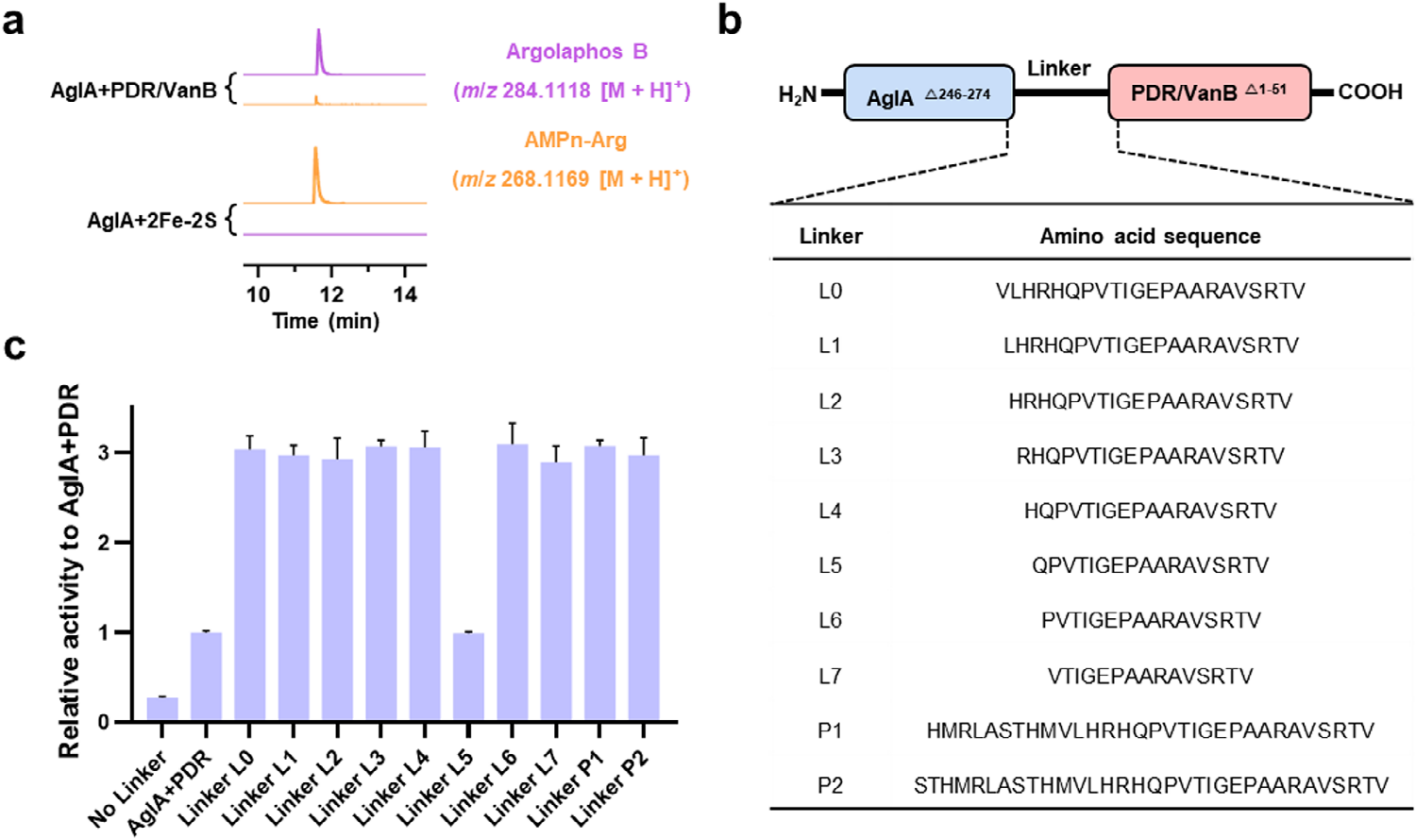
Optimizing AglA as a self‐sufficient biocatalyst. a) LC‒HRMS analysis of the redox partner required for AglA catalysis. The extracted ion chromatograms revealed AMPn‐Arg (orange, *m*/*z* 268.1169 [M + H]⁺) conversion to argolaphos B (purple, *m*/*z* 284.1118 [M + H]⁺) with PDR/VanB family oxidoreductase but not the 2Fe‐2S cluster protein. b) Design of self‐sufficient artificial chimeric enzymes. AglA was fused to PDR/VanB oxidoreductase via ten different peptide linkers of varying lengths and compositions (sequences shown in the table), eliminating the need for separate electron transfer proteins. c) Comparative hydroxylation activities of the chimeric enzyme constructs. The relative activities highlight linker optimization for electron transfer efficiency. Data from technical triplicate (n = 3) were used to calculate and display the average ± SD of each variant. SD, standard deviation. Error bars, SD.

To increase the utility of AglA as a biocatalyst, we created a self‐sufficient, single‐polypeptide system by fusing AglA to its auxiliary reductase partner. Bioinformatic analysis of the host strain (*Streptomyces monomycini* NRRL B‐24309) harboring the argolaphos B biosynthesis‐related gene cluster identified two candidate reductases: a 2Fe‐2S cluster protein and a PDR/VanB family oxidoreductase (Figure S12, Supporting Information). Both reductases contain the essential domains (NAD(P)H‐binding, FAD/FMN‐binding, and Fe‐S cluster‐binding) needed to transfer electrons to AglA's heme. In vitro assays revealed that only the PDR/VanB reductase supported AglA‐catalyzed N^ε^‐hydroxylation of AMPn‐Arg (**Figure** **6**a).

Truncation of the AglA C‐terminus and systematic testing of ten artificial linkers^[^ ^32^ ^]^ enabled successful construction of AglA–PDR/VanB chimeras (Figure 6b, Figure S13a, Supporting Information). Most linkers and their corresponding chimeras demonstrated higher activity than the parental two‐component system (and the unfused two‐component proteins, respectively), with the AglA‐L6‐PDR/VanB chimera—incorporating linker L6—not only exhibiting the highest turnover rate but also showing a 2.8‐fold increase in *k*_cat_/*K*_m_ compared with the unfused two‐component proteins (Figure 6c; Figure S13b Table S5, Supporting Information). One linker (L5) unexpectedly reduced activity, indicating the requirement for fine‐tuned interdomain geometry—a principle consistent with other self‐sufficient P450 designs.^[^ ^32^, ^33^ ^]^

The success of the AglA‐L6‐PDR/VanB chimera demonstrates the feasibility of embedding redox partners within the YqcI/YcgG family. By optimizing reductase selection and linker design, it is possible to create streamlined, self‐sufficient variants with improved catalytic performance, paving the way for the industrial‐scale synthesis of guanidine‐hydroxylated intermediates.

## 3 Discussion

The YqcI/YcgG protein family remains a focal point of scientific interest because of its critical role in the biosynthesis of antimicrobial natural products (NPs).^[^ ^14^, ^15^, ^16^, ^17^, ^19^, ^22^ ^]^ Despite its ubiquity across bacteria, archaea, and eukaryotes (Figure S1, Supporting Information), the lack of detailed structural insights has hindered its industrial exploitation as a biocatalyst for antimicrobial synthesis. Here, we address this gap by revealing the structure–function relationships of AglA,^[^ ^17^ ^]^ the first biochemically characterized heme‐dependent hydroxylase within this family. Through integrated crystallographic, mutagenesis, biochemical, and computational approaches, we propose a dynamic regiochemical gating mechanism that governs substrate positioning for N^ε^‐hydroxylation of AMPn‐Arg (Figure 3d).

AglA adopts an unprecedented “sandwich” fold, comprising a central four‐stranded β‐sheet flanked by helices and loops that cradle a thiolate‐bound heme cofactor (Figure 2a). This architecture diverges sharply from the α‐helical frameworks of canonical cytochrome P450 enzymes yet retains a conserved heme‐coordinating cysteine (Cys44), suggesting evolutionary convergence toward analogous catalytic functions. AlphaFold3^[^ ^26^ ^]^ modeling of other YqcI/YcgG members confirmed the conservation of this fold (Figure S4, Supporting Information), underscoring its functional importance. These findings not only delineate the catalytic blueprint of this enzyme family but also highlight the structural versatility of AglA, positioning it as a robust platform for biocatalyst design.

The catalytic efficiency of AglA hinges on an induced‐fit mechanism mediated by a network of polar and hydrophobic interactions. In the AglA‐AMPn‐Arg complex, residues Phe152, Arg179, and Phe182, along with the flexible loop L2, reconfigure to anchor the substrate's backbone and phosphate group, forming a regiochemical gate that precisely aligns the guanidine moiety for N^ε^ hydroxylation (Figure 2d,e). In contrast, free arginine elicits minimal conformational adjustments, leading to suboptimal guanidine positioning and reduced activity (Figure 3b; Figure S6, Supporting Information). Strikingly, single‐site mutations (e.g., K196R/H/M, K225E, and T220R) disrupt this selectivity, enabling the hydroxylation of free arginine at either N^δ^ or N^ω^ (Figure 4). Such plasticity, achieved with minimal perturbations to the distal binding pocket, reveals the enzyme's adaptability and hints at broader substrate promiscuity through targeted engineering.

In addition to mechanistic studies, we demonstrated the practical utility of AglA as a biocatalyst. The fusion of AglA with its native PDR/VanB oxidoreductase into a single polypeptide mimicking self‐sufficient P450 systems^[^ ^33^, ^34^, ^35^ ^]^ dramatically enhances catalytic turnover (**Figure** **6**; Figure S13, Supporting Information). This chimeric design eliminates the need for exogenous redox partners, simplifies the reaction setup, and improves the coupling efficiency between NAD(P)H oxidation and substrate hydroxylation. Optimal activity was observed in the AglA‐L6‐PDR/VanB construct, where linker flexibility likely facilitates interdomain electron transfer. This modular strategy, which is applicable to homologs such as DcsA^[^ ^19^ ^]^ and GntA,^[^ ^22^ ^]^ underscores the potential for engineering redox‐efficient YqcI/YcgG biocatalysts.

Despite these advances, challenges remain. First, engineered variants exhibit suboptimal catalytic efficiency toward free arginine compared with the native substrate, suggesting that further optimization of active‐site or distal residues via directed evolution or computational design is warranted. Second, high‐resolution structural validation (e.g., cryo‐EM) of the AglA‐L6‐PDR/VanB fusion is needed to dissect interdomain electron transfer mechanisms. Third, enzyme engineering enabled AglA to modulate free L‐arginine hydroxylation regioselectivity, providing preliminary evidence of controllable N^δ^/N^ω^ selectivity. However, its substrate scope remains limited: engineered variants neither alter the selectivity of the native substrate AMPn‐Arg nor act on other arginine derivatives. These boundaries outline clear next steps for pocket remodeling and directed evolution. Additionally, exploring substrate flexibility in other family members (e.g., ChmN^[^ ^16^ ^]^) may reveal the evolutionary strategies of guanidine hydroxylation across contexts, and answering these questions will accelerate the biotechnological deployment of YqcI/YcgG enzymes.

## 4 Conclusion

In summary, our study deciphers the structural and mechanistic underpinnings of an unconventional heme‐dependent hydroxylase. AglA's novel fold, catalytic malleability, and successful conversion into a self‐sufficient biocatalyst lay the groundwork for engineering guanidine hydroxylases as versatile tools in natural product synthesis and synthetic biology. Future efforts to increase the substrate scope, catalytic efficiency, and electron transfer dynamics will further unlock the biotechnological potential of YqcI/YcgG enzymes, mirroring the transformative impact of P450s in late‐stage molecular functionalization.

## 5 Experimental Section

### Materials

Oligonucleotide primer synthesis and DNA sequencing were performed by Tsingke (Beijing, China). All the molecular cloning materials used in this study were obtained from Vazyme Biotech Co., Ltd. (Nanjing, China). Spinach ferredoxin, spinach ferredoxin reductase, and tRNA sourced from *E. coli* MRE 600 were purchased from Sigma‒Aldrich. Unless otherwise stated, all other chemicals were supplied by Sangon Biotech Co., Ltd. (Shanghai, China).

### Gene Cloning and Construction of the Recombinant Plasmid

The genes encoding *AglC* (NCBI accession No. URQ58548.1), *AglA* (NCBI accession No. URQ58546.1), *ChmN* (NCBI accession No. WTY20845.1), *DcsA* (NCBI accession No. D2Z024.1), *2Fe‐2S iron‒sulfur cluster‐binding protein* (NCBI accession No.WP_03 001 7807.1), and *PDR/VanB family oxidoreductase* (NCBI accession No. WP_03 001 9846.1), all derived from *Streptomyces monomycini* NRRL B‐24309, were synthesized by Tsingke (Beijing, China) and optimized for expression in *E. coli*. The gene encoding *ArgRS* (NCBI accession No. NP_416 390.1) was amplified via PCR from *E. coli* DH5α *λ‐pir* genomic DNA. All genes were subcloned and inserted into the pET‐21b vector (Novagen, USA) at the *Nde*I and *Xho*I sites, resulting in the addition of a C‐terminal 6×His tag. The truncated PDR/VanB protein (with residues 1–55 deleted) was produced and fused with an N‐terminal glutathione‐S‐transferase (GST) tag via the pGEX‐6P‐1 vector (Novagen, USA). Constructs for self‐sufficient enzyme variants featuring various linkers were cloned and inserted into the pET‐15b vector (Novagen, USA) at the *Nde*I and *Xho*I sites, harboring an N‐terminal 6×His tag. Point mutations in AglA were introduced via quick‐change PCR using the AglA‐pET‐21b plasmid as the template and primers containing the desired base mutations. All the recombinant plasmids were transformed into *E. coli* DH5α cells, which were subsequently verified via DNA sequencing.

### Protein Expression and Purification

*E. coli* BL21 (DE3) cells transformed with plasmids were cultured in Luria broth (LB) medium supplemented with 0.1 g L^−1^ ampicillin at 37 °C until the optical density at 600 nm (OD600) reached 0.6. Protein expression was induced by the addition of 200 µM isopropyl β‐D‐thiogalactopyranoside (IPTG), followed by incubation at 16 °C with shaking at 180 rpm for 16–18 h. To increase the heme content in the purified heme‐dependent enzymes, 1 mM 5‐aminolevulinic acid (Shanghai Yuanye Bio‐Technology Co., Ltd.) and 1 mM ferrous ammonium sulfate were added to the culture medium during induction with IPTG.

The cell pellets were harvested via centrifugation at 4 °C, 3800 rpm for 15 min, and then they were suspended in lysis buffer containing 25 mM Tris‐HCl (pH 7.5), 200 mM NaCl, and 1 mM phenylmethanesulfonyl fluoride (PMSF). The cells were disrupted via a French press (AH‐1500, ATS, China). After lysis, the lysate was centrifuged at 18 000 rpm at 4 °C for 30 min to remove the cell debris and insoluble material. The supernatant containing the 6×His‐tagged proteins was applied to a nickel affinity column (Ni‐NTA; GE Healthcare, UK) and washed with lysis buffer containing 20 and 30 mM imidazole. The target proteins were eluted with lysis buffer containing 250 mM imidazole. GST‐tagged proteins were purified via GST Sepharose resin (GE Healthcare, UK), followed by GST‐tag cleavage with PreScission protease. Further purification was performed via an anion exchange column (Source Q; GE Healthcare, Sweden) and size exclusion chromatography columns (Superdex200 Increase 10/30 GL; GE Healthcare, Sweden) in buffer containing 15 mM Tris‐HCl (pH 7.5) and 100 mM NaCl. Protein purity was assessed by sodium dodecyl sulfate‒polyacrylamide gel electrophoresis (SDS‒PAGE). Proteins were flash‐frozen in liquid nitrogen and stored at –80 °C for subsequent crystallization and biochemical analysis.

### Isothermal Titration Calorimetry (ITC)

Purified AglA protein was thawed and diluted to a final concentration of 0.2 mM with lysis buffer for ITC experiments. AMPn‐Arg or L‐arginine was dissolved in the same buffer for protein dilution to a concentration of 5 mM. The ligand binding affinities of AglA for AMPn‐Arg or L‐arginine were assessed via Nano ITC (Nano ITC‐ Low Volume; TA Instruments, USA). Titrations were performed at 16 °C by injecting 2.5 µL of ligand solution into a sample chamber containing 300 µL of 0.2 mM AglA protein. A total of 20 injections were made at 120‐s intervals with a stirring speed of 200 rpm. The raw data were analyzed via the single‐site binding model and processed with NanoAnalyze software (version 3.8.0).

### Enzymatic Preparation of AMPn‐Arg

The preparation and purification of AMPn‐Arg were conducted as previously reported.^21^ A total reaction volume of 15 mL was prepared, containing 15 mM AMPn, 30 mM ATP, 30 mM L‐arginine, 20 mg of total tRNA sourced from *E. coli* MRE 600, 40 µM ArgRS (dissolved in 50 mM NH_4_HCO_3_, 100 mM NaCl, pH 7.5), 40 µM AglC (dissolved in 50 mM NH_4_HCO_3_, 100 mM NaCl, pH 7.5), 150 mM NaCl, and 2 mM MgCl_2_ in 100 mM NH_4_HCO_3_ (pH 7.5). The mixture was incubated at 25 °C for 3–5 days, during which ArgRS and AglC were replenished as needed to maintain enzymatic activity.

Following the reaction, proteins were removed from the reaction mixture via 3 kDa Amicon Ultra15 centrifugal filters (Millipore Sigma, Darmstadt, Germany). The filtrate was passed over a Dowex 50WX8 cation exchange resin (Shanghai Macklin Biochemical Co., Ltd.) and washed with deionized water until the eluent reached a neutral pH (7.0–8.0). The resin was then eluted with 2% NH_4_OH, and the eluate was adjusted to pH 7.0 via acetic acid. The fractions containing AMPn‐Arg were lyophilized twice to remove excess moisture and dissolved in deionized water for subsequent HPLC purification.

HPLC purification was performed via an XBridge BEH amide chromatographic column (Waters, Milford, MA, USA; 5 µm, 130 Å, and 4.6 × 150 mm). The detection wavelength was set at λ = 210 nm. The mobile phase consisted of deionized water with 0.1% formic acid (solvent A) and acetonitrile with 0.1% formic acid (solvent B). The flow rate was maintained at 0.5 mL min^−1^, and the gradient program was as follows: 70% B for 5 min; a linear decrease from 70% to 30% B over 25 min; 30% B for 5 min; a linear increase from 30% to 70% B over 10 min; and 70% B for an additional 10 min. The injection volume was 10 µL, and the column was equilibrated for 15 min before each injection.

### Crystallization and Structure Determination

Purified AglA and its variants were concentrated to 25 mg mL^−1^ for crystallization. Crystallization screening (Index, Wizard I/II/III/IV, PEG/ION, SaltRX, Crystal; Hampton Research, USA) was performed by sitting‐drop vapor‐diffusion assays at 16 °C. The protein‒ligand complex was prepared by incubating proteins with AMPn‐Arg or L‐arginine at 4 °C for 2‒3 h (molar ratio = 1:5).

Crystals were obtained under various conditions and grown to suitable sizes for X‐ray diffraction within one week. Before data collection, the crystals were cryoprotected with a solution containing 5% glycerol. X‐ray diffraction data were collected at beamline 18U1 of the Shanghai Synchrotron Radiation Facility (SSRF). With an exposure time of 0.2 s and a crystal‐to‐detector distance of 400 mm, a total of 360 images were recorded at 1.0° oscillations. The data were indexed, integrated, and scaled via the XDS software suite^[^ ^36^ ^]^ and CCP4i2.^[^ ^37^ ^]^ The crystal structure of AglA was solved by molecular replacement via a model predicted by AlphaFold2.^[^ ^38^ ^]^ Next, the optimal solution was manually built in COOT and refined in PHENIX.^[^ ^39^, ^40^, ^41^ ^]^ Data collection and processing statistics are included in Table S1 and Table S4 (Supporting Information). The final models were validated via MolProbity and deposited in the Protein Data Bank.

### AglA Enzymatic Activity Assays

Reactions (100 µL) containing 10 µM AglA enzyme, 200 µM AMPn‐Arg or L‐arginine, 5 µg spinach ferredoxin, 0.02 U spinach ferredoxin reductase, 1 mM NAD(P)H, and 25 mM HEPES buffer (pH 7.5) were incubated at 25 °C for 1 h. Methanol (100 µL) was added to precipitate AglA, and the reaction mixtures were placed on ice for 15 min before centrifugation at 12,000 rpm for 15 min at 4 °C. The reaction products were collected for subsequent LC–HRMS/MS analysis. The catalytic activity of AglA is reflected by integrating the peak areas of the extracted ion chromatograms in LC‐HRMS.

### Mass Spectrometry

Mass spectrometry analysis was performed via an Ultimate 3000 rapid separation liquid chromatograph coupled with a Q Exactive Plus Q‐Orbitrap HRMS (Thermo Fisher Scientific, Waltham, MA, USA). The HESI source parameters were as follows: sheath gas at 35 arb, auxiliary gas at 10 arb, spray voltage at 3.5 kV for positive ion mode and 3.2 kV for negative ion mode, capillary temperature at 320 °C, auxiliary gas heater temperature at 350 °C, and s‐lens RF level at 60%. The mass data were acquired in data‐dependent MS/MS (ddMS2) mode via the following parameters: *m/z* range from 60–900, mass resolution, 70000; AGC target, 3e6; maximum IT, 100 ms; *m/z* isolation window within 1.6 Da, intensity threshold to trigger MS/MS acquisition, 1e5; dynamic exclusion, 5 s; and stepped NCE at 15, 30, 45 eV, TopN = 8. Separation was conducted on a BEH amide column (Waters, Milford, MA, USA; 2.1×100 mm, 1.7 µm) at a flow rate of 0.3 mL min^−1^. The oven temperature was maintained at 40 °C. Extracted ion chromatograms (EICs) were generated via Xcalibur software (Thermo Fisher Scientific) with a 10 ppm error range.

AMPn‐Arg and argolaphos B were analyzed in positive mode. The mobile phase consisted of solvent A (deionized water) and solvent B (acetonitrile: deionized water, 90:10, v/v), both of which contained 0.1% formic acid. The gradient was as follows: 0–2 min, 100% B; 2–10 min, linear decrease from 100% to 60% B; 10–18 min, linear decrease from 60% to 45% B; 18–19 min, linear increase from 45% to 100% B; and 19–30 min, re‐equilibration at 100% B.

L‐arginine and N‐hydroxyarginine were analyzed in negative mode. The mobile phase was composed of solvent A (deionized water) and solvent B (acetonitrile‐deionized water, 90:10, v/v), both of which contained 10 mM ammonium formate and 0.15% formic acid. The gradient was as follows: 0–2 min, 100% B; 2–9 min, linear decrease from 100% to 85% B; 9–14 min, linear decrease from 85% to 50% B; 14–19 min, 50% B; and 19–27 min, re‐equilibration at 100% B.

### Calculation of the Binding Free Energy Change (ΔΔG) for Single‐Site Saturation Mutagenesis of AglA

On the basis of the resolved crystal structure of the AglA‐AMPn‐Arg complex, virtual saturation mutagenesis was performed on eighteen active‐site residues, including those in loop L2 and the substrate‐binding region, to identify variants that might enhance interactions with free L‐arginine. The crystal structure of heme‐bound AglA served as the receptor input in AutoDock v4.2.0.^[^ ^42^ ^]^ The L‐arginine ligand was prepared in PDBQT format via the prepare_ligand4.py script. The active‐site box, defined to encompass the substrate‐binding pocket with dimensions of 20 Å in x, y, and z, was used for docking, as this size was determined to effectively cover the potential interaction region on the basis of preliminary structural analysis. By employing the Lamarckian genetic algorithm, ten docking conformations were generated, with the lowest‐energy conformation selected for further analysis.

To ensure high accuracy in mutational ΔΔG analysis, the Rosetta suite's cartesian_ddg protocol^[^ ^43^ ^]^ was utilized. Virtual saturation mutagenesis was conducted at the target sites identified from the docking results. Mutations were evaluated on the basis of calculated ΔΔG_binding values (defined as ΔG_mutant – ΔG_wildtype), where negative values indicate increased binding affinity. Finally, the top eight most energetically favorable mutations were selected. These mutations guided subsequent site‐directed mutagenesis experiments on the original residues, ensuring a systematic and targeted approach to identifying critical functional residues.

### Molecular Dynamics Simulation

Molecular dynamics simulations were conducted via GROMACS 2021.7 software.^[^ ^44^ ^]^ Charmm36‐jul2022.ff^[^ ^45^ ^]^ was employed for amino acid residues, whereas the TIP3P model^[^ ^46^ ^]^ was utilized for solvent water molecules. The protonation states of all the titratable residues were determined on the basis of the pKa values predicted by the H++ server,^[^ ^47^ ^]^ considering the hydrogen bonding network. The parameters for the heme‐Fe(IV) = O species were taken from a previous study.^[^ ^48^ ^]^

Initial structures of AglA variants were predicted via AlphaFold3^[^ ^26^ ^]^ and subsequently docked with the heme‐Fe(IV) = O intermediate and L‐arginine via AutoDock v4.2.0.^[^ ^42^ ^]^ The resulting complexes were immersed in a cubic solvent box, ensuring a minimum distance of 30 Å between the protein surface and the box edges. The systems were neutralized by adding appropriate amounts of Na⁺ and Cl^−^ ions.

Energy minimization was performed in two stages: the 5000‐step steepest descent method to remove unreasonable contacts, followed by 5000 steps of the conjugate gradient. The system was subsequently heated from 0 K to 300 K at 100 ps via the NVT ensemble with a constraint constant of 50 kcal mol^−1^ Å^2^ for the protein complex. This was followed by 500 ps NPT ensemble equilibration at 1 bar pressure and 300 K, with a 5 kcal mol^−1^ Å^2^ restraint constant. Production simulations with a duration of 200 ns were conducted via the NPT ensemble without any constraints. Trajectory analysis was conducted via VMD 1.9.3 software,^[^ ^49^ ^]^ which focuses on root mean square deviation (RMSD) to assess protein stability and flexibility.

### Bioinformatic Analysis

PF08892‐associated protein sequences were systematically curated from the Pfam^[^ ^50^ ^]^ (https://pfam.xfam.org) and InterPro^[^ ^51^ ^]^ (https://www.ebi.ac.uk/interpro) databases through programmatic access. Multiple sequence alignment was executed via MAFFT v7.526^[^ ^52^ ^]^ under the L‐INS‐i algorithm optimized for local homology detection, with 10 refinement iterations for precision enhancement. The maximum likelihood phylogeny was reconstructed via FastTree v2.1^[^ ^53^ ^]^ implementing the JTT+CAT evolutionary model with gamma rate heterogeneity, which was supported by 1000 ultrafast bootstrap pseudoreplicates. The tree topology was validated via the SH‐like approximate likelihood ratio test before visualization refinement through iTOL v7.2^[^ ^54^ ^]^ supplemented with the itol.toolkit v0.1.6 R^[^ ^55^ ^]^ package for batch annotation processing. De novo tertiary structures of representative homologs (Mhr24, ChmN, GntA, DcsA, AmbT) were predicted through AlphaFold3^[^ ^26^ ^]^ with five model replications, retaining the highest pLDDT‐scored conformations (>90). Consensus sequence features were determined via ClustalW v2.1 pairwise alignment via a BLOSUM62 substitution matrix with gap penalties of 10/0.1 (open/extended). Evolutionarily conserved motifs were mapped through ESPript 3.0^[^ ^56^ ^]^ with a 90% sequence similarity threshold and secondary structure elements superimposed from reference PDB templates (AglA).

### Steady‐State Enzyme Kinetic Assays

The steady‐state kinetic parameters were calculated according to the substrate consumed via LC‐HRMS technology. First, standard samples of the substrates AMPn‐Arg and L‐arginine with a concentration range of 0.001–2 mM were prepared, and standard curves relating the concentration of the two compounds to the peak area of the ion peaks were established, ensuring that the correlation coefficient R^2^ > 0.98.

The total volume of the reaction system was 100 µL, containing purified recombinant protein of chimeric enzymes, 1 mM NAD(P)H, and different concentrations of the substrate AMPn‐Arg or L‐arginine in 15 mM HEPES buffer (pH 7.5). The above reaction system (without enzyme) was preincubated at 25 °C for 5 min, and then, the enzyme was added to initiate the reaction; moreover, a reaction mixture without enzyme was used as the blank control. After 10 min of reaction, 5 volumes of ice‐cold methanol were added to terminate the reaction. The reaction mixture was centrifuged, and the supernatant was collected, freeze‐dried, and then reconstituted with 100 µL of ultrapure water. The supernatant was collected again after centrifugation for LC‐HRMS detection. The difference between the ion peak area of the substrate in the blank control group and that in the reaction group was the peak area corresponding to the substrate consumed by the enzyme‐catalyzed reaction, and the amount of substrate consumed was calculated via combination with the standard curve. Nonlinear regression analysis was performed via GraphPad Prism v. 10.0.3 software to calculate the steady‐state kinetic parameters of the enzymatic reaction.

### Statistical Analysis

In this study, the catalytic activity of all relevant enzymes was determined and calculated via LC‐HRMS technology. The peak area corresponding to the target compound was integrated to obtain raw data, which were then subjected to subsequent processing. For calculation, the peak area corresponding to the target compound in the control group was taken as the reference (set to 1). The corresponding ion peak area of each treatment group was subsequently subjected to ratio calculation with this reference value, and the final result obtained was the relative enzymatic catalytic activity. All the quantitative data are expressed as the means ± standard deviations (SDs) unless otherwise specified. All the assays were performed in technical triplicate (n = 3).

## Conflict of Interest

The authors declare no conflict of interest.

## Author Contributions

Y.S., C.D., and W.Y. contributed equally to this work. X. Z. and W. C. designed the research. Y. S. and W. Y. generated the constructs, purified the proteins, and performed the ITC and activity assays. Y. S. and W. Y. grew and optimized the crystals. C. D. and Y. Z. collected the data. C. D. determined the structure. P.C. executed the molecular dynamics simulation. Y. S. and L. Z. examined the enzyme activity and performed the mass spectrometry. W. C., X. Z., C. D., Y. S., W. Y., D. Z., Z. L., S. L., X. X., and Q. H. analyzed the data. W. Y., C. D., X. Z., and W. C. wrote the manuscript with contributions from other authors.

## Supporting information



Supporting Information

## Data Availability

The coordinates and structure factors of apo‐AglA, AglA‐Arg, AglA‐AMPn‐Arg, AglAK196R‐Arg, AglAK225E‐Arg, and AglAT220R‐Arg have been deposited at the RCSB Protein Data Bank with accession numbers 9UB3, 9UB5, 9UBT, 9UBA, 9UBB and 9UBS, respectively. All other data are available in the main text or Supplementary information.
