# Supplementary material for: A Dynamic Gate Enables Regioselective Hydroxylation of Free Arginine by a Non‐Canonical Heme Enzyme
Source: Adv Sci (Weinh). 2025 Nov 12;13(6):e13032. doi: 10.1002/advs.202513032 (PMC12866846; doi:10.1002/advs.202513032)
Supplement: Supplementary file 1 — Supporting Information [file ADVS-13-e13032-s001.docx]

**Supporting Information**

**A Dynamic Gate Enables Regioselective Hydroxylation of Free Arginine by a Non-Canonical Heme Enzyme**

*Yuan Sun, Chao Dou, Weizhu Yan, Pengpeng Chen, Lu Zhang, Dan Zhou, Yanhui Zheng, Zhaolin Long, Shoujie Li, Xiaoqing Xu, Qiuxia Huang, Xiaofeng Zhu*, Wei Cheng**

Y. Sun, W. Yan, P. Chen, D. Zhou, Y. Zheng, Z. Long, S. Li, X. Xu, Q. Huang, X. Zhu, W. Cheng

Department of Pulmonary and Critical Care Medicine, Respiratory Infection and Intervention Laboratory of Frontiers Science Center for Disease-related Molecular Network, and State Key Laboratory of Biotherapy, West China Hospital of Sichuan University, Chengdu, 610041, China.

Email: zhuxiaofeng@scu.edu.cn; chengwei669@scu.edu.cn

C. Dou, W. Cheng

Antibiotics Research and Re-evaluation Key Laboratory of Sichuan Province, Sichuan Industrial Institute of Antibiotics, School of Pharmacy, Chengdu University, Chengdu 610106, China.

L. Zhang

Metabolomics and Proteomics Technology Platform of Core Facilities, West China Hospital, Sichuan University, Chengdu, 610041, China.

Yuan Sun, Chao Dou, and Weizhu Yan contributed equally to this work.

**Supplementary Figures**
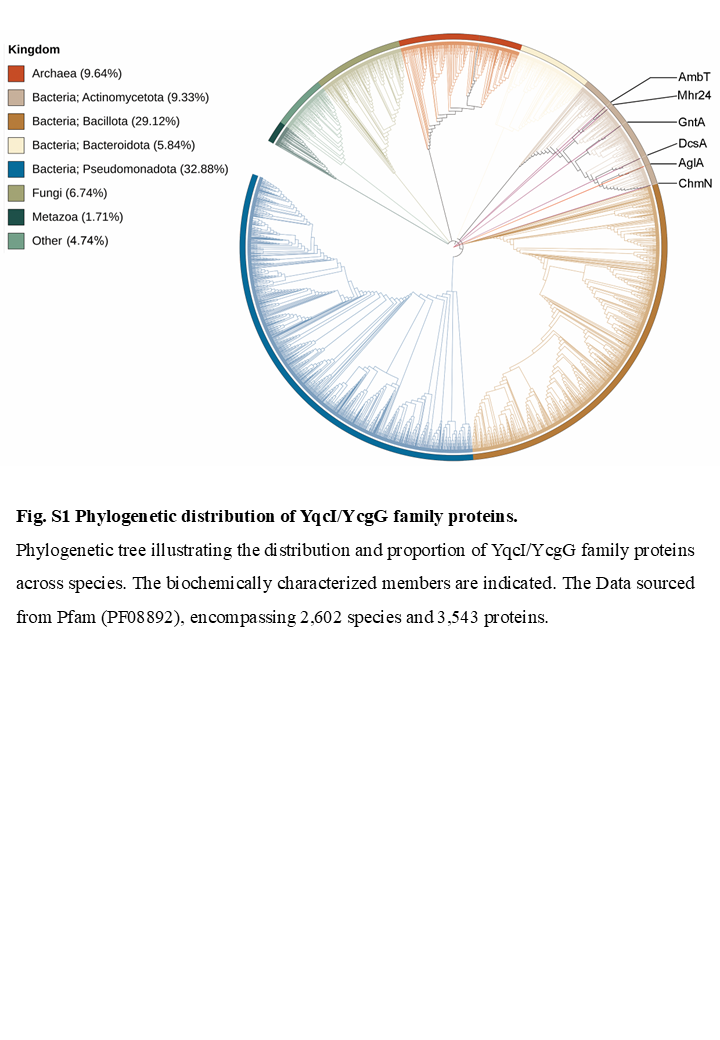


**Fig. S1 Phylogenetic distribution of YqcI/YcgG family proteins.**

Phylogenetic tree illustrating the distribution and proportion of YqcI/YcgG family proteins across species. The biochemically characterized members are indicated. The Data sourced from Pfam (PF08892), encompassing 2,602 species and 3,543 proteins.

**
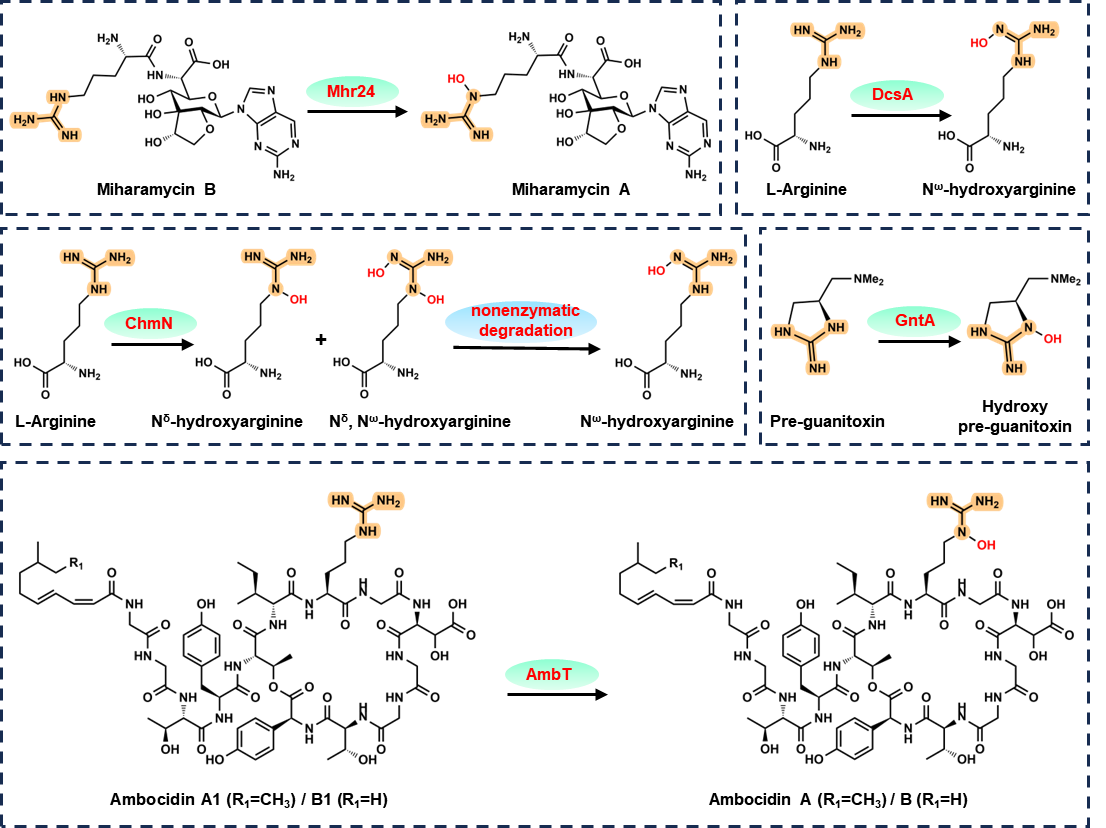
Fig. S2 The catalytic reactions mediated by YqcI/YcgG family enzymes.**

Overview of characterized reactions catalyzed by YqcI/YcgG family members, showcasing the functional versatility in guanidine N–H hydroxylation of this enzyme superfamily.

**
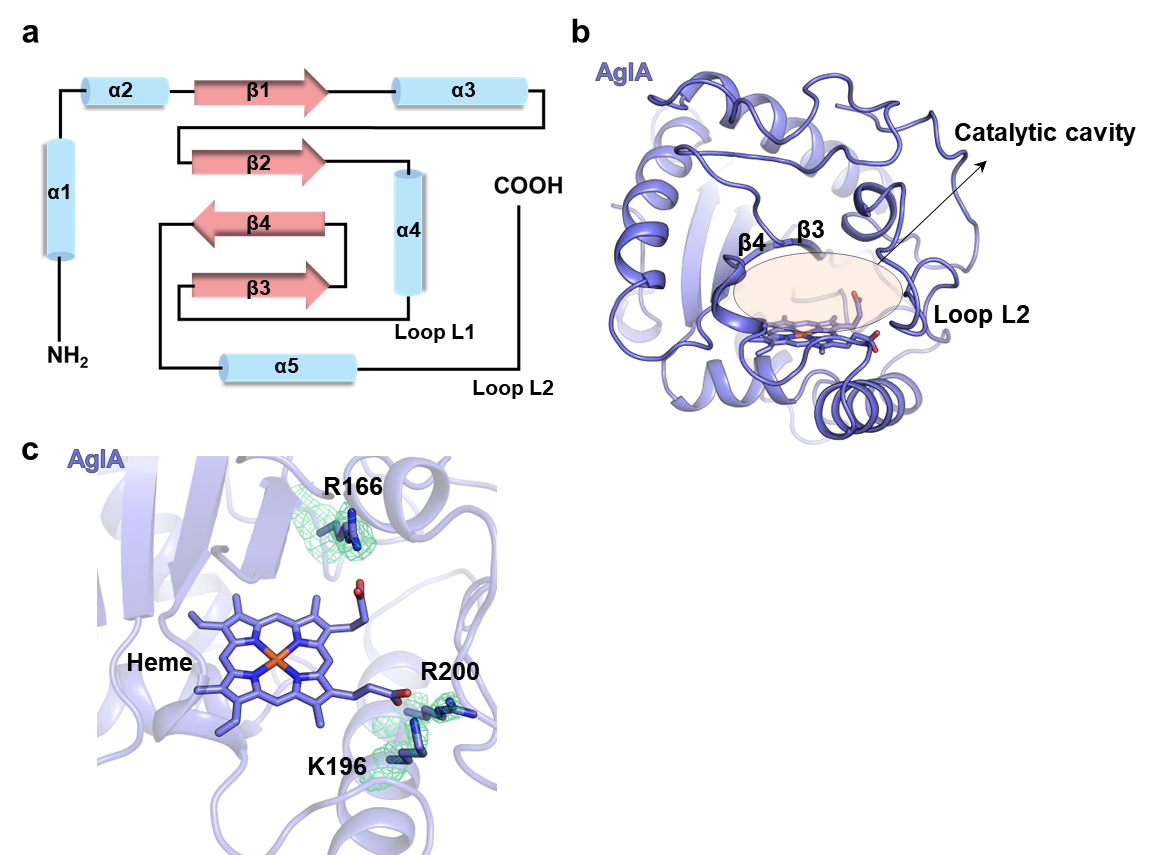
Fig. S3 Topological architecture and catalytic cavity organization of AglA.**

**(a)** Topological diagram of AglA, with α-helices (blue cylinders) and β-strands (red arrows) depicting the “sandwich” fold. **(b)** The catalytic cavity above heme is bounded by β3, β4, and loop L2, facilitating substrate binding and hydroxylation. **(c)** The omit map for the key residues (R166, R200, K196) (contoured at 3.0σ).


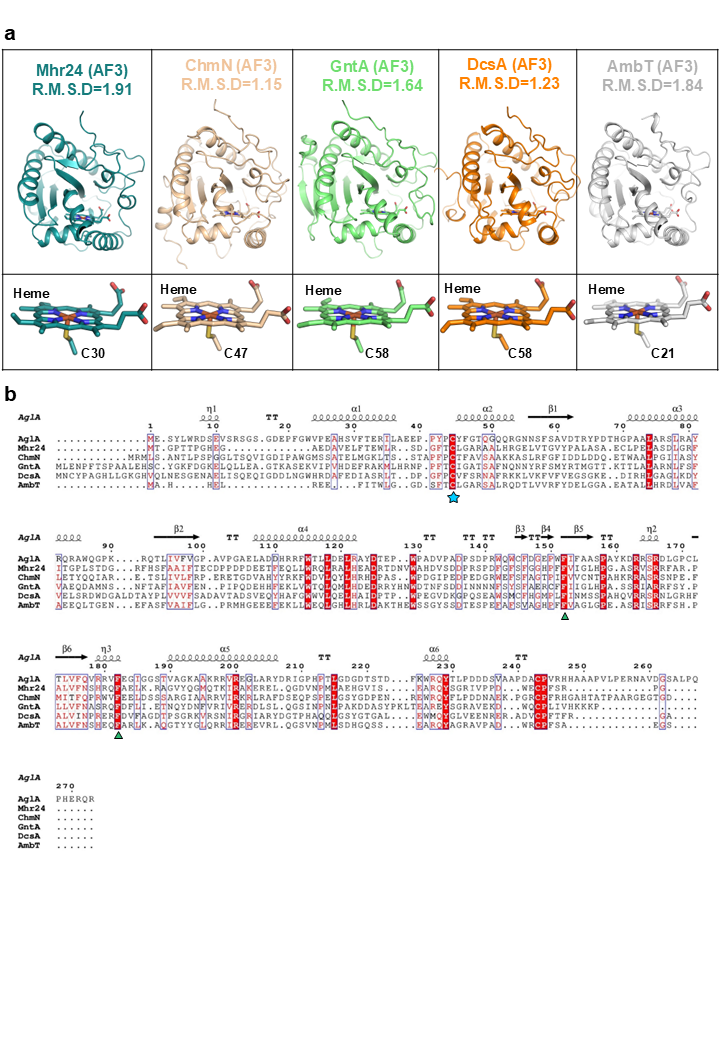


**Fig. S4 Structural conservation in the YqcI/YcgG family.**

**(a)** AlphaFold3-predicted structures of YqcI/YcgG family members (Mhr24, ChmN, GntA, DcsA, and AmbT) aligned with AglA, sharing the core fold and a conserved heme-proximal cysteine. RMSDs relative to apo-AglA are shown. **(b)** Sequence alignment of AglA with homologs Mhr24 (QCX41930.1, 23.9% similarity), ChmN (WTY20845.1, 43.7%), GntA (WP_096685072.1, 33.4%), DcsA (D2Z024.1, 37.3%), and AmbT (WP_079030457.1, 28.2%). Conserved residues are in red, with heme-binding cysteine (blue pentagram) and conserved phenylalanine residue (green triangle) marked.

**
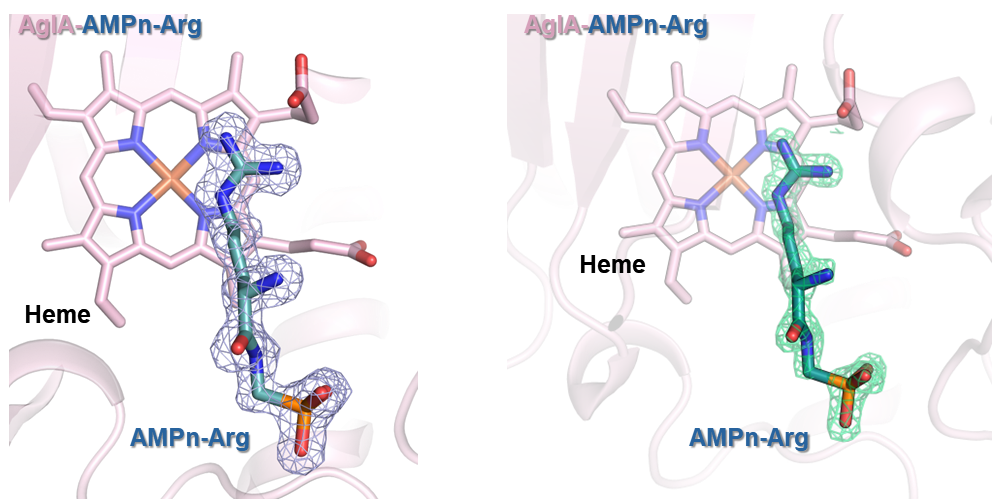
**

**Fig. S5 Electron density of AMPn-Arg.**

2Fo-Fc electron density map (contoured at 1.0σ, left), omit map (contoured at 3.0σ, right) showing the bound AMPn-Arg substrate in the AglA-AMPn-Arg complex structure, confirming the precise positioning of the substrate in the enzyme active site.

**
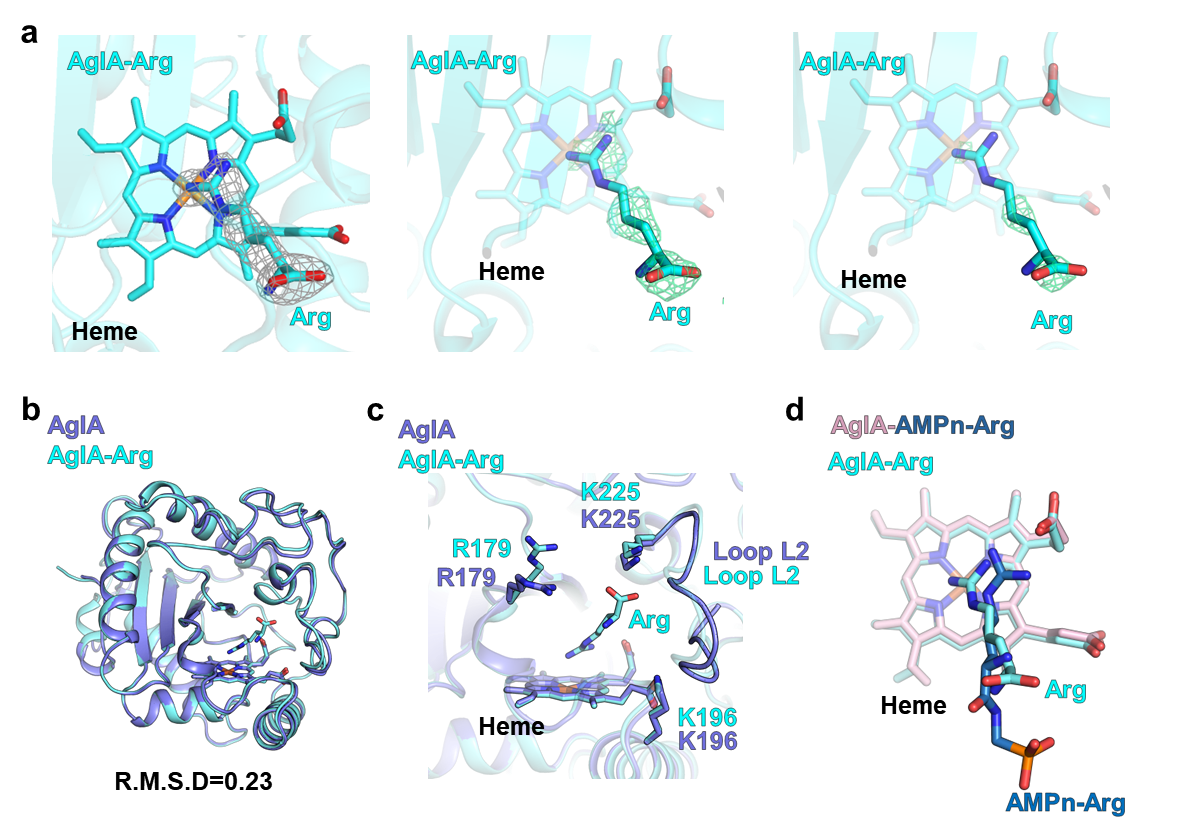
Fig. S6 Structural comparison of apo AglA with AglA-Arg and AglA-AMPn-Arg.**

**(a)** 2Fo-Fc electron density map (contoured at 1.0σ, left), omit map (contoured at 2.0σ, middle), and omit map (contoured at 3.0σ, right) showing arginine binding in the AglA-Arg complex. **(b)** Superposition of apo AglA and AglA-Arg structures. **(c)** Close-up view of Lys225, Lys196, and Arg179 residues in apo AglA and AglA-Arg, demonstrating their inability to form polar contacts with the arginine backbone in the AglA-Arg complex. **(d)** Arginine binding in AglA-Arg, with the guanidinium N^ω^ atom oriented toward the heme’s distal axial site, contrasting with AMPn-Arg’s N^δ^ orientation.

**
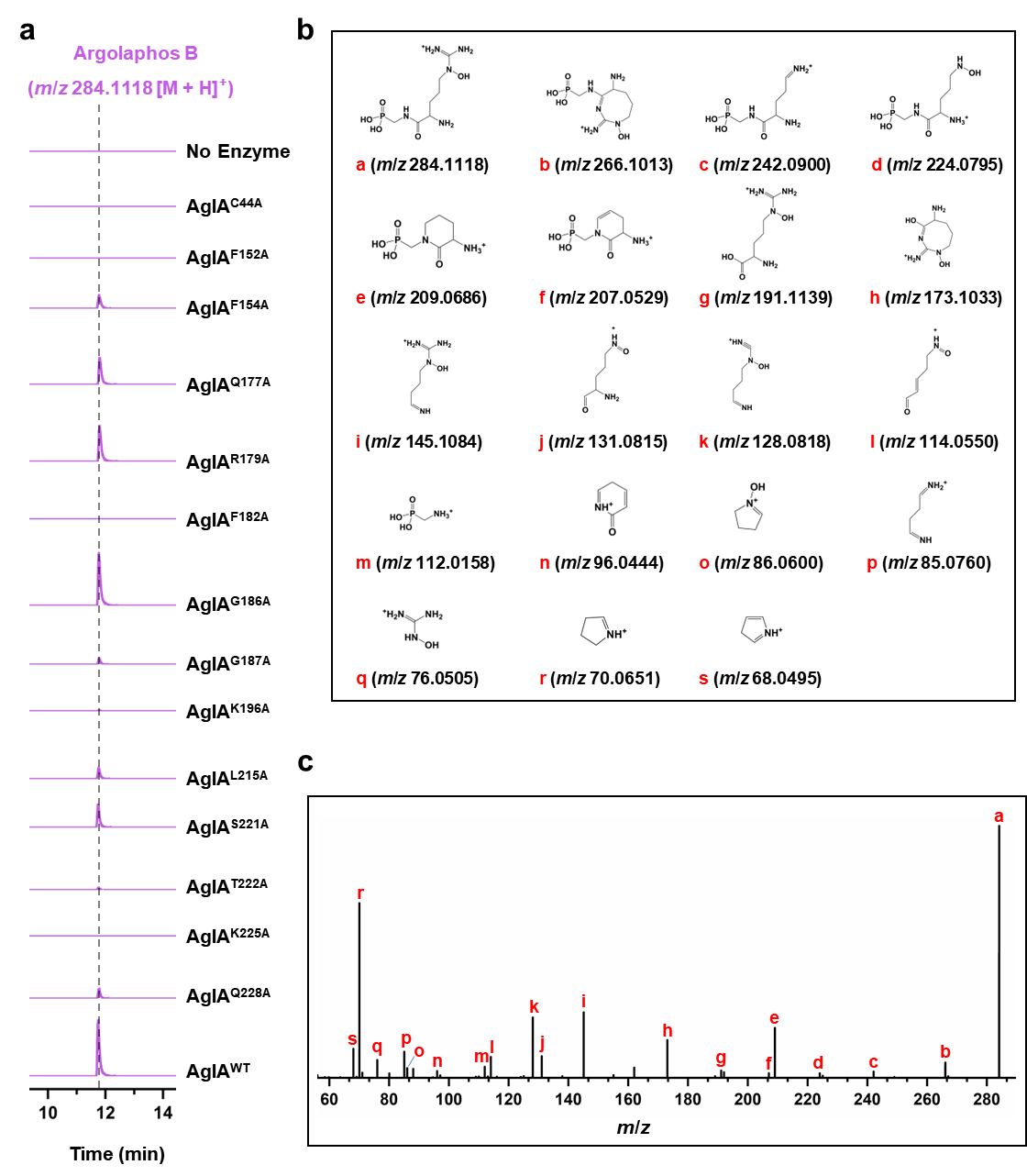
Fig. S7 Mutational analysis of AglA activity toward AMPn-Arg and characterization of argolaphos B.**

**(a)** LC–HRMS analysis of AglA variants’ activity toward AMPn-Arg, with chromatograms showing argolaphos B formation (*m*/*z* 284.1118 [M + H]⁺). **(b)** LC‒HRMS/MS spectrum and fragmentation pattern of argolaphos B, confirming its structure. **(c)** Detailed LC‒HRMS/MS spectrum of argolaphos B produced by wild-type AglA.

**
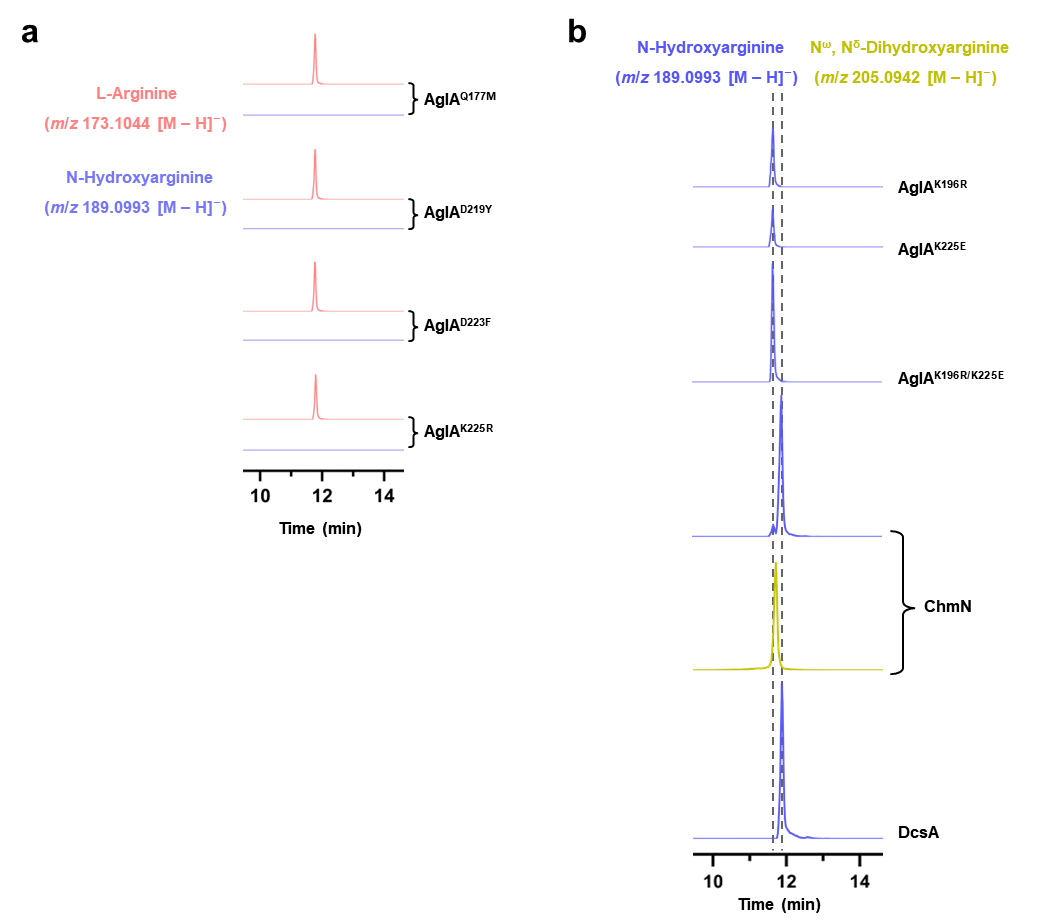
Fig. S8** **Effect of active site mutations on AglA-catalyzed arginine hydroxylation.**

**(a)** LC‒HRMS analysis of the catalytic activity of AglA^Q177M^, AglA^D219Y^, AglA^D223F^ and AglA^K225R^ towards arginine. Extracted ion chromatograms show arginine (pink, *m*/*z* 173.1044 [M – H]⁻ and hydroxyarginine (slate blue, *m*/*z* 189.0993 [M – H]⁻). **(b)** Comparative activity analysis of AglA^K196R^, AglA^K225E^ and AglA^K196R/K225E^, ChmN, DcsA toward free arginine, with hydroxyarginine product formation (*m*/*z* 189.0993 [M – H]⁻) indicated by slate blue, and dihydroxyarginine product formation (*m*/*z* 205.0942 [M – H]⁻)indicated by dark yellow extracted ion chromatograms.


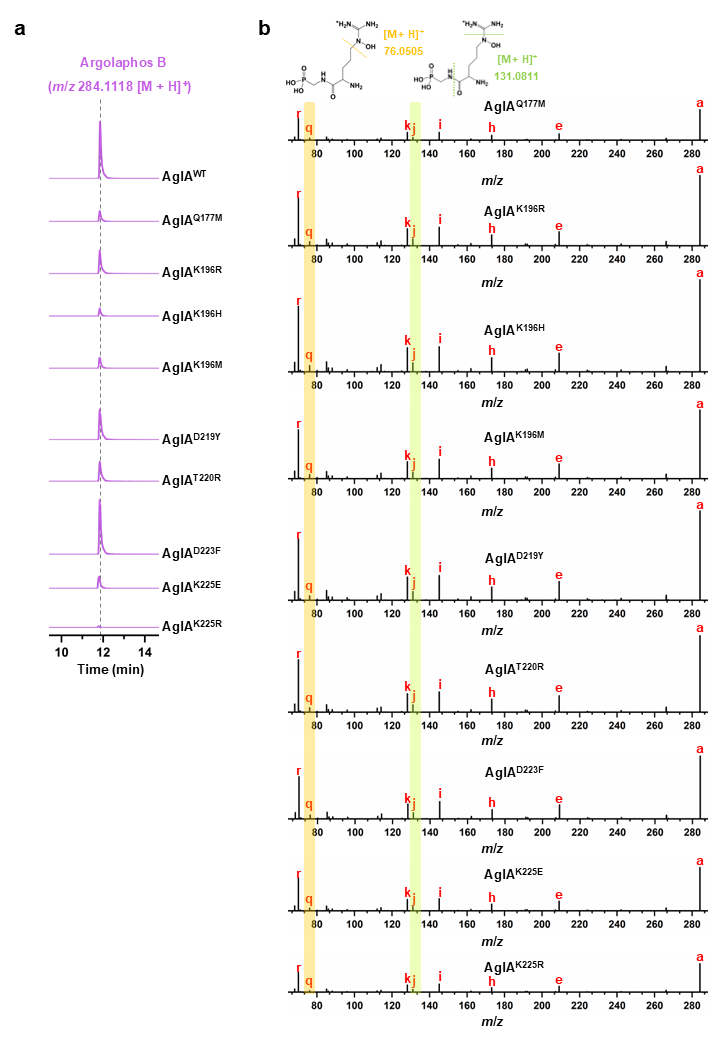
**Fig. S9 Impact of engineered AglA variants on AMPn-Arg hydroxylation.**

**(a)** LC‒HRMS analysis of AglA variants’ activity toward AMPn-Arg, with extracted ion chromatograms showing argolaphos B (*m*/*z* 284.1118 [M + H]⁺). **(b)** LC‒HRMS/MS of argolaphos B produced by different variants, with diagnostic fragments (*m*/*z* 76.0505 [M + H]⁺ and *m*/*z* 131.0811 [M + H]⁺) confirming N^ε^-hydroxylation.

**
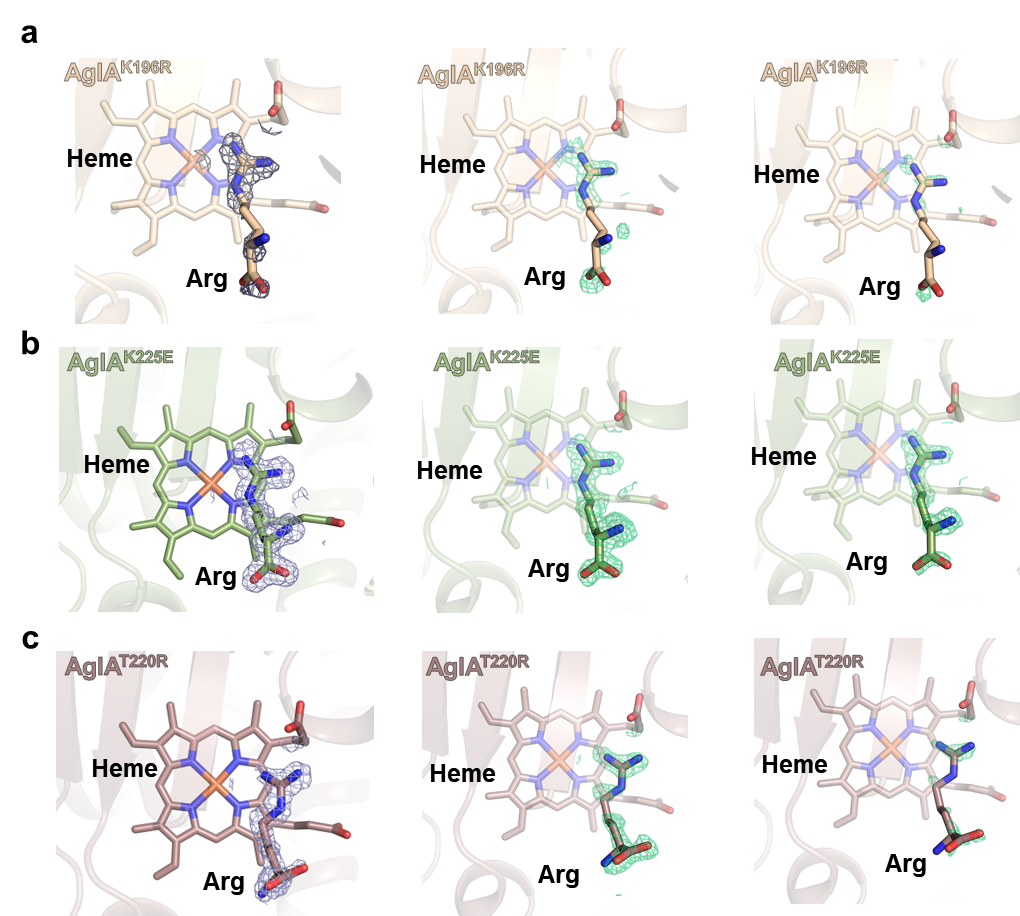
Fig. S10 Electron density for arginine binding in engineered AglA variants.**

**(a)** 2Fo-Fc electron density map (contoured at 1.0σ, left), omit map (contoured at 2.0σ, middle), and omit map (contoured at 3.0σ, right) showing arginine binding in the AglA^K196R^-Arg complex. **(b)** 2Fo-Fc electron density map (contoured at 1.0σ, left), omit map (contoured at 2.0σ, middle), and omit map (contoured at 3.0σ, right) showing arginine binding in the AglA^K225E^-Arg complex. **(c)** 2Fo-Fc electron density map (contoured at 1.0σ, left), omit map (contoured at 2.0σ, middle), and omit map (contoured at 3.0σ, right) showing arginine binding in the AglA^T220R^-Arg complex.


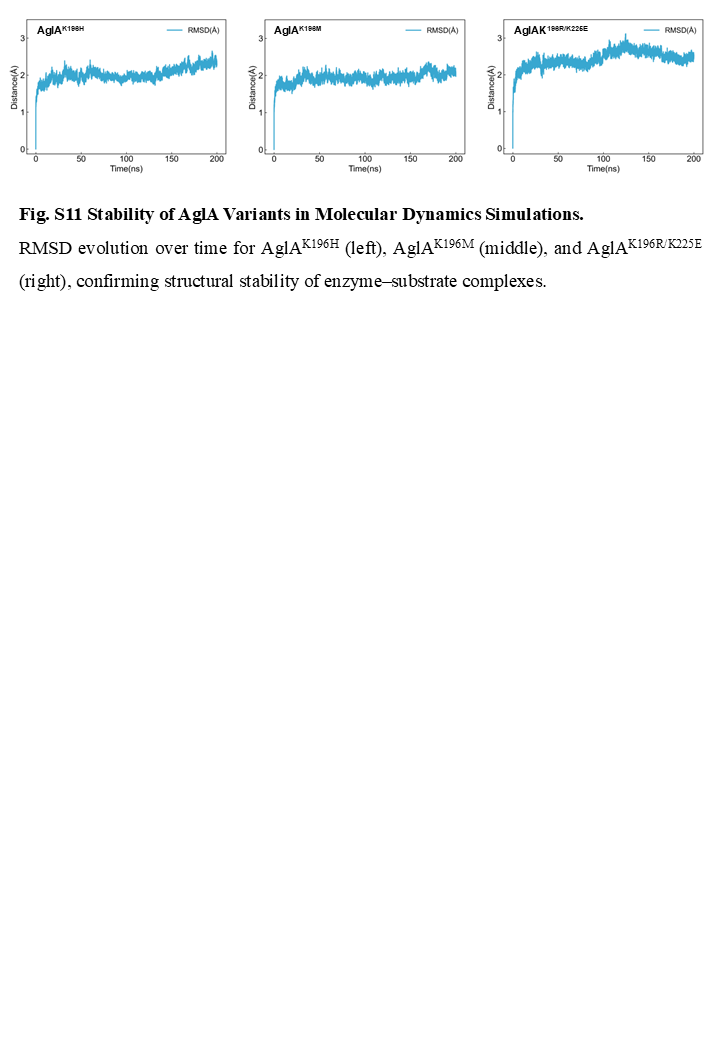
**Fig. S11 Stability of AglA Variants in Molecular Dynamics Simulations.**

RMSD evolution over time for AglA^K196H^ (left), AglA^K196M^ (middle), and AglA^K196R/K225E^ (right), confirming structural stability of enzyme–substrate complexes.


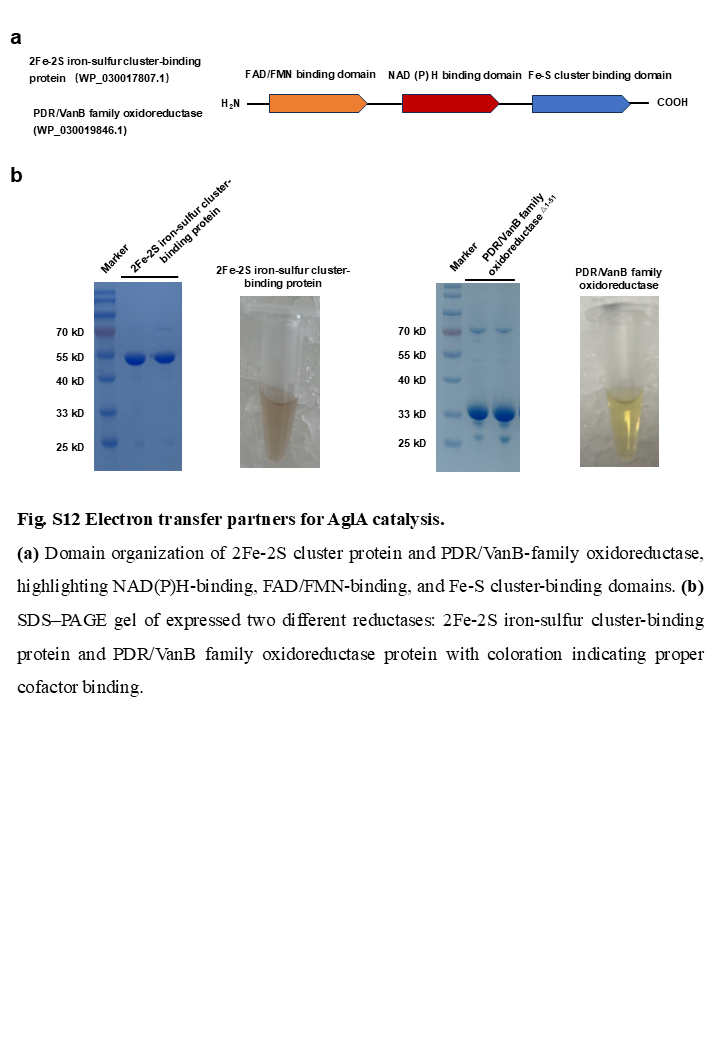
**Fig. S12 Electron transfer partners for AglA catalysis.**

**(a)** Domain organization of 2Fe-2S cluster protein and PDR/VanB-family oxidoreductase, highlighting NAD(P)H-binding, FAD/FMN-binding, and Fe-S cluster-binding domains. **(b)** SDS‒PAGE gel of expressed two different reductases: 2Fe-2S iron-sulfur cluster-binding protein and PDR/VanB family oxidoreductase protein with coloration indicating proper cofactor binding.

**
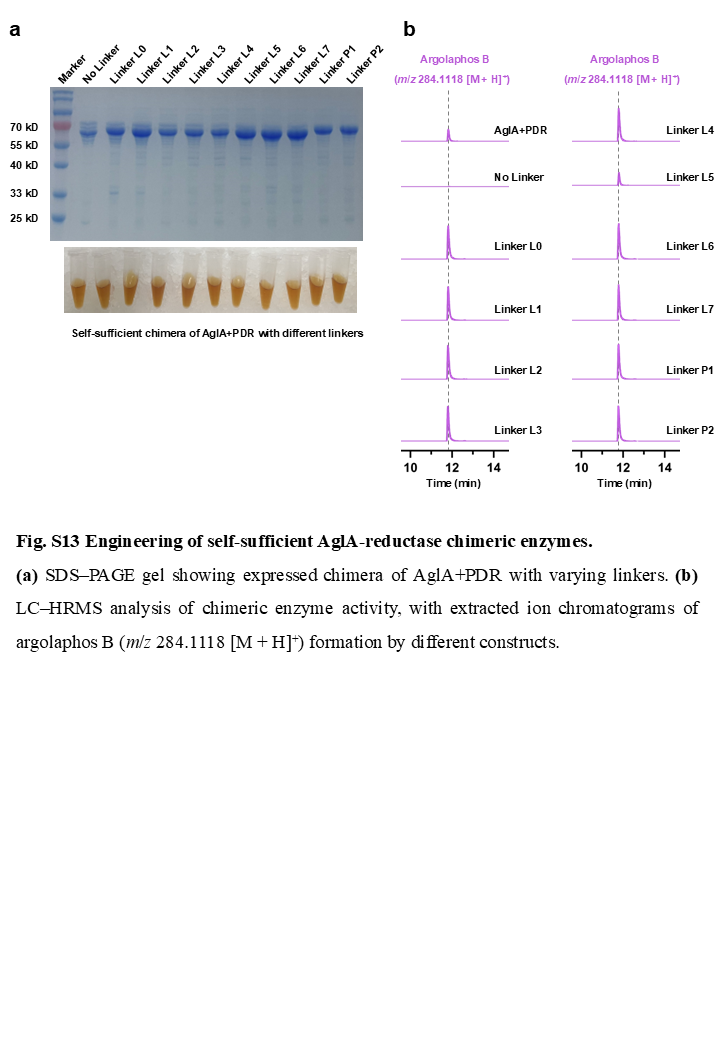
Fig. S13 Engineering of self-sufficient AglA-reductase chimeric enzymes.**

**(a)** SDS‒PAGE gel showing expressed chimera of AglA+PDR with varying linkers. **(b)** LC‒HRMS analysis of chimeric enzyme activity, with extracted ion chromatograms of argolaphos B (*m*/*z* 284.1118 [M + H]⁺) formation by different constructs.

**Table S1. Data collect and refinement statistics**

|  | **apo-AglA**  **PDB ID: 9UB3** | **AglA-Arg**  **PDB ID:** **9UB5** | **AglA-AMPn-Arg**  **PDB ID:** **9UBT** |
| --- | --- | --- | --- |
| **Data collect** |  |  |  |
| Space group | P 4_3_ 2_1_ 2 | P 4_3_ 2_1_ 2 | P 2_1_ 2_1_ 2_1_ |
| **Cell dimensions** |  |  |  |
| a, b, c (Å) | 77.251, 77.251, 116.086 | 76.460, 76.460, 99.690 | 48.168, 72.221, 75.385 |
| α, β, γ (^o^) | 90.00, 90.00, 90.00 | 90.00, 90.00, 90.00 | 90.00, 90.00, 90.00 |
| **Resolution (Å)** | 34.60 - 2.06  (2.15 - 2.06) | 36.65 - 1.83  (1.90 - 1.83) | 40.07 - 1. 60  (1.64 - 1.60) |
| ***R*_merge_ (%)** | 10.92 | 9.82 | 16.15 |
| ***R*_pim_ (%)** | 6.07 | 2.08 | 6.77 |
| ***I/σ* (*I*)** | 13.50 (2.50) | 19.04 (2.75) | 6.16 (0.91) |
| **Completeness (%)** | 99.92 (99.96) | 100.00 (100.00) | 99.74 (98.83) |
| **Redundancy** | 3.3 (1.1) | 24.0 (26.5) | 6.4 (4.2) |
| **Refinement** |  |  |  |
| **No. reflections** | 22532 (2767) | 26739 (2611) | 35593 (2877) |
| ***R*_work_/*R*_free_ (%)** | 17.37/21.99 | 18.21/22.31 | 17.55/19.70 |
| **No. atoms** | 2044 | 2204 | 2281 |
| Protein | 1938 | 1959 | 1964 |
| Ligand | 43 | 43 | 43 |
| solvent | 63 | 202 | 274 |
| **Average B-factors** | 48.68 | 36.49 | 12.44 |
| **Ramachandran favored (%)** | 98.76 | 98.35 | 98.35 |
| **Ramachandran allowed (%)** | 1.24 | 1.65 | 1.65 |
| **Ramachandran outliers (%)** | 0.00 | 0.00 | 0.00 |
| **R.m.s deviations** |  |  |  |
| Bond length (Å) | 0.013 | 0.016 | 0.013 |
| Bond angles (^o^) | 1.89 | 2.20 | 1.68 |

**Statistics for the highest-resolution shell are shown in parentheses**

**Table S2. Homologous structure search results of AglA on the DALI server**

| **PDB** | **Z Score** | **RMSD** | **Description** |
| --- | --- | --- | --- |
| 6NEQ-Z | 5.4 | 5.1 | 28S Ribosomal RNA, Mitochondrial |
| 5LMN-X | 5.2 | 3.5 | 16S Ribosomal RNA |
| 3I4T-A | 5.0 | 4.0 | Diphthine Synthase |
| 3V97-A | 5.0 | 3.5 | Ribosomal RNA Large Subunit Methyltransferase L |
| 6W6V-F | 4.9 | 4.2 | Yeast RNase MRP holoenzyme |
| 9O0D-B | 4.7 | 4.9 | rSAMD9 tRNase |
| 3EVZ-A | 4.6 | 3.4 | Methyltransferase |
| 9UIE-B | 4.6 | 3.5 | RNA (32-MER) |
| 8XDO-A | 4.6 | 3.5 | Norbelladine O-Methyltransferase |
| 1WDE-A | 4.5 | 3.8 | Probable Diphthine Synthase |

**Table S3. Enzymatic kinetic parameters for the mutants of AglA catalyzing substrate L-arginine**

| **Enzyme** | ***k*_cat_ (min^−1^)** | ***K*_m_ (μM)** | ***k*_cat_/*K*_m_ (s^−1^ M^−1^)** |
| --- | --- | --- | --- |
| AglA^K196R^ | 2.05 ± 0.07 | 77.48 ± 11.93 | 441 |
| AglA^K225E^ | 1.18 ± 0.12 | 54.94 ± 8.77 | 358 |
| AglA^K196R/K225E^ | 2.15 ± 0.06 | 42.68 ± 8.78 | 840 |
| AglA^K196H^ | 1.94 ± 0.08 | 66.99 ± 13.05 | 483 |
| AglA^K196M^ | 0.84 ± 0.07 | 42.59 ± 7.61 | 329 |
| AglA^T220R^ | 1.13 ± 0.13 | 42.03 ± 6.46 | 448 |

Reaction conditions: purified recombinant protein of chimeric enzymes, 1 mM NADPH, different concentrations of the substrate L-Arginine in 15 mM HEPES Buffer (pH 7.5), 25 °C for 10 min.

**Table S4. Data collect and refinement statistics**

|  | **AglA^K196R^-Arg**  **PDB ID: 9UBA** | **AglA^K225E^-Arg**  **PDB ID: 9UBB** | **AglA^T220R^-Arg**  **PDB ID: 9UBS** |
| --- | --- | --- | --- |
| **Data collect** |  |  |  |
| Space group | P 2_1_ 2_1_ 2_1_ | P 2_1_ 2_1_ 2_1_ | P 2_1_ 2_1_ 2_1_ |
| **Cell dimensions** |  |  |  |
| a, b, c (Å) | 47.910, 73.123, 75.086 | 48.000, 73.310, 74.930 | 47.974, 72.394, 75.197 |
| α, β, γ (^o^) | 90.00, 90.00, 90.00 | 90.00, 90.00, 90.00 | 90.00, 90.00, 90.00 |
| **Resolution (Å)** | 37.54 - 1.47  (1.51 - 1.47) | 21.82 - 1.41  (1.45 - 1.41) | 36.20 - 1.49  (1.53 - 1.49) |
| ***R*_merge_ (%)** | 11.42 | 12.12 | 6.10 |
| ***R*_pim_ (%)** | 5.56 | 3.49 | 2.49 |
| ***I/σ* (*I*)** | 7.51 (0.26) | 13.04 (2.19) | 19.29 (2.53) |
| **Completeness (%)** | 98.99 (97.55) | 99.98 (100.00) | 96.80 (94.26) |
| **Redundancy** | 4.7 (1.1) | 12.9 (12.5) | 7.0 (6.8) |
| **Refinement** |  |  |  |
| **No. reflections** | 45144 (3146) | 51661 (3617) | 42108 (2859) |
| ***R*_work_/*R*_free_ (%)** | 17.65/20.02 | 18.09/21.21 | 16.78/19.07 |
| **No. atoms** | 2270 | 2356 | 2307 |
| Protein | 1919 | 1940 | 1934 |
| Ligand | 43 | 43 | 43 |
| solvent | 308 | 373 | 330 |
| **Average B-factors** | 18.71 | 19.12 | 19.34 |
| **Ramachandran favored (%)** | 98.72 | 98.31 | 98.31 |
| **Ramachandran allowed (%)** | 1.28 | 1.69 | 1.69 |
| **Ramachandran outliers (%)** | 0.00 | 0.00 | 0.00 |
| **R.m.s deviations** |  |  |  |
| Bond length (Å) | 0.008 | 0.009 | 0.011 |
| Bond angles (^o^) | 1.01 | 1.05 | 1.16 |

**Statistics for the highest-resolution shell are shown in parentheses**

**Table S5. Enzymatic kinetic parameters for the self-sufficient AglA-reductase chimeric enzyme that catalyzes the substrate AMPn-Arg**

| **Enzyme** | ***k*_cat_ (min^−1^)** | ***K*_m_ (μM)** | ***k*_cat_/*K*_m_ (s^−1^ M^−1^)** |
| --- | --- | --- | --- |
| AglA^WT^ | 4.35 ± 0.09 | 67.89 ± 7.22 | 1068 |
| AglA-PDR No Linker | 4.72 ± 0.10 | 86.73 ± 9.08 | 907 |
| AglA-PDR Linker L0 | 4.55 ± 0.09 | 41.85 ± 5.36 | 1830 |
| AglA-PDR Linker L1 | 4.54 ± 0.09 | 32.86 ± 3.94 | 2304 |
| AglA-PDR Linker L2 | 5.60 ± 0.16 | 35.56 ± 4.76 | 2624 |
| AglA-PDR Linker L3 | 6.04 ± 0.12 | 42.79 ± 5.37 | 2353 |
| AglA-PDR Linker L4 | 6.11 ± 0.11 | 39.17 ± 4.38 | 2600 |
| AglA-PDR Linker L5 | 5.94 ± 0.19 | 88.46 ± 11.71 | 1119 |
| AglA-PDR Linker L6 | 6.07 ± 0.14 | 34.25 ± 4.35 | 2955 |
| AglA-PDR Linker L7 | 4.84 ± 0.12 | 46.84 ± 6.64 | 1722 |
| AglA-PDR Linker P1 | 6.57 ± 0.14 | 49.47 ± 6.64 | 2213 |
| AglA-PDR Linker P2 | 5.64 ± 0.13 | 39.68 ± 5.36 | 2369 |

Reaction conditions: purified recombinant protein of chimeric enzymes, 1 mM NADH, different concentrations of the substrate AMPn-Arg in 15 mM HEPES Buffer (pH 7.5), 25 °C for 10 min.

**Table S6. List of primers used in this study**

| **Name** | **Forward primer** | **Reverse primer** |
| --- | --- | --- |
| pET21b-AglC | TTTAACTTTAAGAAGGAGATATACATATGATCGGTACCCGTACC | TCTCAGTGGTGGTGGTGGTGGTGCTCGAGACGACGTACTGCTG |
| pET21b-ArgRS | TTTAACTTTAAGAAGGAGATATACATATGAATATTCAGGCTCTTCTCTCAG | ATCTCAGTGGTGGTGGTGGTGGTGCTCGAGCATACGCTCTACAGTCTCAATAC |
| pET21b-2Fe-2S | TTTAACTTTAAGAAGGAGATATACATATGTTCCATCCATTGCG | TCTCAGTGGTGGTGGTGGTGGTGCTCGAGACGGTCGAAATCCAGT |
| pGEX-PDR/VanB^Δ1-51^ | TCCAGGGGCCCCTGGGATCCGGTCCACTTCACCTGC | TCACGATGCGGCCGCTCGAGTCACAGGTCCAGTACCAGAC |
| pET21b-AglA^C44A^ | CAGAAGAACCGCCGTATCCGGCTTATTTTG | CTGACCCTGTGTACCAAAATAAGCCGGATACG |
| pET21b-AglA^F152A^ | GTTTTGATGGTGAACCGTGGGCTATTTTTG | CGGAGAGGCTGCAAAAATAGCCCACG |
| pET21b-AglA^F154A^ | GTGAACCGTGGTTTATTGCTGCAGCC | ATGCCGGAGAGGCTGCAGCAATAAAC |
| pET21b-AglA^F182A^ | TTCAGGTTCGTCGTGTTGCTGAAGG | CTACCACCAATACCTTCAGCAACACG |
| pET21b-AglA^G186A^ | CGTCGTGTTTTTGAAGGTATTGCTGGTAG | CCGCAACAGTACTACCAGCAATACC |
| pET21b-AglA^G187A^ | CGTCGTGTTTTTGAAGGTATTGGTGCTAGTAC | TTTACCCGCAACAGTACTAGCACCAAT |
| pET21b-AglA^K196A^ | GCGGGTAAAGCAGCAGCACGTC | CTTCACGAACACGACGTGCTGCTG |
| pET21b-AglA^K225A^ | CGAGCACCGATTTTGCATGGCG | CAGTGTATACTGGCGCCATGCAAAAT |
| pET21b-AglA^L215A^ | GTCCTCATCCTACCGCGGGTGA | CGTATCACCATCACCCGCGGTAG |
| pET21b-AglA^Q228A^ | CACCGATTTTAAATGGCGCGCGTATAC | CATCCGGCAGTGTATACGCGCGC |
| pET21b-AglA^R179A^ | GACACTGGTTTTTCAGGTTGCTCGTG | CAATACCTTCAAAAACACGAGCAACCTG |
| pET21b-AglA^S221A^ | GGTGATGGTGATACGGCCACCGAT | GCGCCATTTAAAATCGGTGGCCGTATC |
| pET21b-AglA^T222A^ | GTGATGGTGATACGAGCGCCGATTT | GCGCCATTTAAAATCGGCGCTCG |
| pET21b-AglA^Q177M^ | GTGTCTGACACTGGTTTTTATGGTTCGTCG | CCAATACCTTCAAAAACACGACGAACCATAAA |
| pET21b-AglA^K196R^ | GGGTAAAGCAGCACGTCGTCG | CTTCACGAACACGACGACGTG |
| pET21b-AglA^K196H^ | GGGTAAAGCAGCACATCGTCGTG | CTTCACGAACACGACGATGTGCTG |
| pET21b-AglA^K196M^ | CGGGTAAAGCAGCAATGCGTC | TCACGAACACGACGCATTGCT |
| pET21b-AglA^D219Y^ | ACCCTGGGTGATGGTTATACGAGCA | GCCATTTAAAATCGGTGCTCGTATAACCATC |
| pET21b-AglA^T220R^ | CCTGGGTGATGGTGATCGTAGCAC | CCATTTAAAATCGGTGCTACGATCACCAT |
| pET21b-AglA^D223F^ | ATGGTGATACGAGCACCTTTTTTAAATGGC | CAGTGTATACTGGCGCCATTTAAAAAAGGTG |
| pET21b-AglA^K225E^ | CGAGCACCGATTTTGAATGGCG | GTGTATACTGGCGCCATTCAAAATCGG |
| pET21b-AglA^K225R^ | CGAGCACCGATTTTCGTTGGCG | GCAGTGTATACTGGCGCCAACGAAAA |
| pET15b-AglA-PDR/VanB-No Linker | TCCGGTTGGTCCACTTCACCTGCT | AGTGGACCAACCGGACATGCATCC |
| pET15b-AglA-PDR/VanB-Linker L0 | ACCGGATGCATGTCCGGTTGTGCTGCACCGTCACCAGCCAGTTACCATCGGTGA | AGCAGGTGAAGTGGACCAACGGTACGAGAAACAGCACGAGCAGCCGGTTCACCGATGGTA |
| pET15b-AglA-PDR/VanB-Linker L1 | ACCGGATGCATGTCCGGTTCTGCACCGTCACCAGCCAGTTACCATCGGTGAACCGGC | AACCAGCAGGTGAAGTGGACCAACGGTACGAGAAACAGCACGAGCAGCCGGTTCACCG |
| pET15b-AglA-PDR/VanB-Linker L2 | CACCGGATGCATGTCCGGTTCACCGTCACCAGCCAGTTACCATCGGTGAACCGGCTGC | CAGCAGGTGAAGTGGACCAACGGTACGAGAAACAGCACGAGCAGCCGGTTCACCGATG |
| pET15b-AglA-PDR/VanB-Linker L3 | CACCGGATGCATGTCCGGTTCGTCACCAGCCAGTTACCATCGGTGAACCGGCTGCT | CAGCAGGTGAAGTGGACCAACGGTACGAGAAACAGCACGAGCAGCCGGTTCACCGA |
| pET15b-AglA-PDR/VanB-Linker L4 | ACCGGATGCATGTCCGGTTCACCAGCCAGTTACCATCGGTGAACCGGCTGCTCGTG | AACCAGCAGGTGAAGTGGACCAACGGTACGAGAAACAGCACGAGCAGCCGGTTCAC |
| pET15b-AglA-PDR/VanB-Linker L5 | CGGATGCATGTCCGGTTCAGCCAGTTACCATCGGTGAACCGGCTGCTCGTGC | CAGCAGGTGAAGTGGACCAACGGTACGAGAAACAGCACGAGCAGCCGGTTCA |
| pET15b-AglA-PDR/VanB-Linker L6 | CACCGGATGCATGTCCGGTTCCAGTTACCATCGGTGAACCGGCTGCTCGT | AGCAGGTGAAGTGGACCAACGGTACGAGAAACAGCACGAGCAGCCGGTTCAC |
| pET15b-AglA-PDR/VanB-Linker L7 | CACCGGATGCATGTCCGGTTGTTACCATCGGTGAACCGGCTGCTCG | GGTGAAGTGGACCAACGGTACGAGAAACAGCACGAGCAGCCGGTTCAC |
| pET15b-AglA-PDR/VanB-Linker P1 | CGGATGCATGTCCGGTTCACATGCGTCTGGCGTCTACCCATATGGTGCTGCA | ATGGTAACTGGCTGGTGACGGTGCAGCACCATATGGGTAGACGCCAGA |
| pET15b-AglA-PDR/VanB-Linker P2 | ACCGGATGCATGTCCGGTTTCTACTCACATGCGTCTGGCGAGCACTCACATGGT | GGTAACTGGCTGGTGACGGTGCAGCACCATGTGAGTGCTCGCCAGACGCATG |
